# Physico-Chemical Alternatives in Lignocellulosic Materials in Relation to the Kind of Component for Fermenting Purposes

**DOI:** 10.3390/ma9070574

**Published:** 2016-07-15

**Authors:** Alberto Coz, Tamara Llano, Eva Cifrián, Javier Viguri, Edmond Maican, Herbert Sixta

**Affiliations:** 1 Green Engineering and Resources, Department of Chemistry and Process and Resource Engineering, University of Cantabria, Avda. Los Castros s/n, Santander 39005, Spain; tamara.llano@unican.es (T.L.); eva.cifrian@unican.es (E.C.); javier.viguri@unican.es (J.V.); 2 Faculty of Biotechnical Systems Engineering, Politehnica University of Bucharest, 313 Splaiul Independentei, Sector 6, Bucuresti 060042, Romania; edmond.maican@upb.ro; 3 Department of Forest Products Technology, School of Chemistry, Aalto University, P.O. Box 16300, Aalto FI-00076, Finland; herbert.sixta@aalto.fi

**Keywords:** biorefinery, fermentation, detoxification, lignocellulosic materials, inhibitors, fractionation

## Abstract

The complete bioconversion of the carbohydrate fraction is of great importance for a lignocellulosic-based biorefinery. However, due to the structure of the lignocellulosic materials, and depending basically on the main parameters within the pretreatment steps, numerous byproducts are generated and they act as inhibitors in the fermentation operations. In this sense, the impact of inhibitory compounds derived from lignocellulosic materials is one of the major challenges for a sustainable biomass-to-biofuel and -bioproduct industry. In order to minimise the negative effects of these compounds, numerous methodologies have been tested including physical, chemical, and biological processes. The main physical and chemical treatments have been studied in this work in relation to the lignocellulosic material and the inhibitor in order to point out the best mechanisms for fermenting purposes. In addition, special attention has been made in the case of lignocellulosic hydrolysates obtained by chemical processes with SO_2_, due to the complex matrix of these materials and the increase in these methodologies in future biorefinery markets. Recommendations of different detoxification methods have been given.

## 1. Introduction

Lignocellulosic materials represent one of the most promising sources of renewable raw material for various biotechnological processes, giving useful biobased chemicals and fuels, due to their low economic value and high availability [1,2,3,4]. Lignocellulosic biomass is the most abundant renewable biological resource and it is outside the human food chain, making it an attractive raw material for biorefinery options. Within lignocellulosic biomass, wood, straw, stalks, and bagasse have a global inventory of 1750, 1145, 970, and 75 million tons of biofibres, respectively [5]. In Europe, a wide variety of biomasses can be found, with European forestry and agriculture highly diversified with a good mix between forest (42% of the European area) and agriculture (40%, i.e., 1.7 Mkm^2^), except in Scandinavia where forest is predominant (up to 70% of forest area) [6].

Lignocellulosic biomass includes herbaceous crops, agriculture and industrial residues such as sugarcane bagasse, corn stover or straw, softwood, hardwood, and municipal solid waste [7,8,9,10]. The choice of raw material depends on location and availability among other factors [11]. For example, in the case of using lignocellulosic waste materials for the conversion of bioethanol, wheat straw (with a production of 354.34 × 10^6^ ton/year), rice straw (731.3 × 10^6^ ton/year), corn straw (128.02 × 10^6^ ton/year), and sugarcane bagasse (180.73 × 10^6^ ton/year) are the four major agro-wastes to be used, according to Sarkar et al. [12].

Lignocellulosic biomass has a complex structure consisting of three major fractions: cellulose (35%–50% dry weight), hemicelluloses (15%–35%), and lignin (10%–25%) [13,14,15,16,17,18]; and their relative abundances depend on the type of biomass feedstock [4,19]. Cellulose is the most abundant natural polymer. It is a linear/linear helical water-insoluble polysaccharide consisting of glucose monomers from several hundred up to tens of thousands [20]. Hemicelluloses are heteropolysaccharides and have an amorphous structure [20], comprising pentoses (xylose, arabinose) and hexoses (mannose, glucose, galactose), which can be substituted with phenolic, uronic, or acetyl groups [10]. Lignin has a complex phenolic polymeric structure. Its structure results from the condensation of phenylpropene units. The precursors of lignin are *p*-hydroxyphenol alcohol, guaïacyl alcohol, and syringyl alcohol. Lignin plays an important role in cell wall structure, acting as a glue holding together the cellulose and hemicellulose fibres [13].

All of the fractions from lignocellulosic biomass can be used to obtain several products. Cellulose has been used as potential production of pulp and/or paper from the year 105 in ancient China. Hemicellulose makes up the second most principal fraction of the plant cell wall after cellulose and is a potential substrate for the production of bioethanol and/or value-added products of commercial significance [4,21,22]. In addition, different products can be obtained from lignin [23,24]: (i) power, fuel, and syngas products; (ii) macromolecules; and (iii) low-molecular weight aromatic or phenolic compounds. However, the heterogeneous nature of polymeric lignin makes it very difficult to control and standardise the properties and quality of lignin products [24]. Despite significant efforts in the past few decades, the commercial lignin market has stagnated at between 1.0 and 1.3 million metric tonnes per year [25]. ‘You can make anything out of lignin…except money’ has long been a myth in the pulp and paper industry. Although this may be an unfair statement, it expresses a deep frustration with over a century’s effort and expectation on lignin product development. The majority of the existing lignin products nowadays are based on lignosulphonates [24].

Due to the possibilities of obtaining a great variety of products and fuels, a new concept—biorefinery—has been developed. A biorefinery is an analogue to the current petro-refinery, in the sense that it produces energy and chemicals. The IEA Bioenergy Task 42 defines biorefining as ‘the sustainable processing of biomass into a spectrum of bio-based products (food, feed, chemicals and/or materials) and bioenergy (biofuels, power and/or heat)’ [26,27] and this term was recently redefined in the project Biorefinery Euroview as follows: “Biorefineries could be described as integrated biobased industries using a variety of technologies to make products such as chemicals, biofuels, food and feed ingredients, biomaterials, fibres and heat and power, aiming at maximising the added value along the three pillars of sustainability (Environment, Economy and Society)” [6].

Conversion of lignocellulosic materials to higher value products requires separation of the material into its components. Pretreatments range from simple size reduction to more advanced biological or physico-chemical processes designed to improve the digestibility of the biomass [28]. Physical, chemical, and biological treatments such as acid or alkaline hydrolysis, enzymatic hydrolysis, solvent extraction, precipitation, membrane technologies and steam, and CO_2_ or ammonia explosion can be used [14,16,29,30,31,32]. Among these processes, thermo-chemical or hydrolysis processes have been recognised as the most extensive processing steps in lignocellulosic biomass to obtain fermentable sugar and other byproducts and several review documents provide a general overview of the field [14,33]. However, due to the heterogeneous nature of lignocellulosic biomass, in addition to sugars, the chemical hydrolysis of lignocellulosics can release several compounds that act as microbial inhibitors [11,22,34]. The type of lignocellulosic material (grasses, hardwoods, softwoods, etc.), the cell wall composition, and the severity of the thermochemical conditions employed for hydrolysis (defined as the combination of time, high temperature, and low pH used) mostly determine the nature of the inhibitors and the concentration can vary greatly [8,22,35,36,37,38,39,40,41,42,43]. Moreover, individual inhibitors may not have a strong effect on fermenting microorganisms, but combinations of them can drastically hamper fermentation reactions [41,44,45,46,47,48,49].

To minimise the negative effects of these compounds on fermentation, numerous methodologies have been tested for hydrolysate treatment, including physical (evaporation), chemical (solvent extraction, overliming, activated charcoal adsorption, ion exchange, etc.), and biological (microbiology, enzyme, adaption of fermenting microorganism, etc.) methods [3,4,11,14,35,37,50,51,52,53,54,55,56,57,58,59,60]. Biological methods (such as laccase- or peroxidase-mediated methods, changes in fermentation strategies, microbial acclimatization, direct implication of microorganisms favouring inhibitor metabolism, and microbial pretreatment of lignocellulose) could be more useful. However, enzymatic detoxification, modified fermentation strategies, and microbial pretreatment of lignocellulose are slow and time-consuming and some of the enzymes are expensive [4,11]; therefore, a lot of research still needs to be carried out on the development and optimisation of these procedures. Furthermore, much of the research has been carried out at laboratory scale and there are few pilot-scale or full-scale investigations on the use of enzymes to detoxify lignocellulosic hydrolysate [11]. On the other hand, physical and physico-chemical methods are fast and better known at pilot scale; however, factors like significant sugar loss, affinities, and cost need to be optimised [22].

The main objective of this review is to analyse the most significant inhibitors in the structure of lignocellulosic materials and the main physical and physico-chemical detoxification methods in order to give some recommendations to valorise the hemicellulosic biomass towards the biorefinery concept. A search of the literature quickly reveals the complicated nature of the topic of hydrolysate toxicity, brought about by the multitude of biomass feedstock, pretreatment and conditioning methods, fermentation methods, and fermentation strains tested. Different biomass feedstocks and pretreatment processes generate different combinations of toxic compounds; different fermentation strains have different levels of natural resistance; and changes in the fermentation processes can lead to different levels of resistance [49]. In order to clarify the obtained results, detoxification processes have been analysed in relation to the group of inhibitors and the raw material. The best results have been obtained and discussed. In addition, novel procedures and the combination of different processes have been studied. Finally, due to the importance of the chemical pretreatments with SO_2_ to depolymerise the lignocellulosic materials into a high-purity-cellulose and the production of lignosulphonates in the hydrolysates, a section about the possibilities of detoxification processes in these kinds of materials has been added in this review.

## 2. Inhibitors in Lignocellulosic Materials

**Figure 1:**
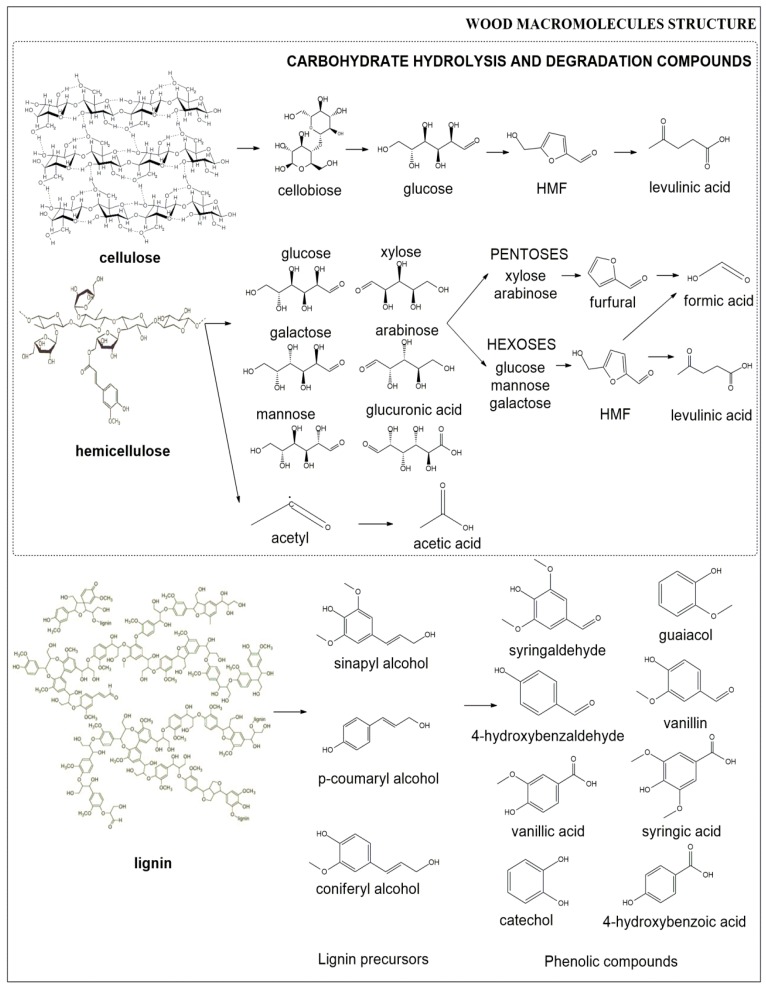
Inhibitors in lignocellulosic materials.

The classification of the inhibitors is based mainly on the origin and they can be divided into the following major groups: furan derivatives such as furfural and 5-hydroxymethylfurfural (HMF), phenolic compounds, weak organic acids (levulinic, formic, and acetic acids), raw material extractives (acidic resins, tannic acids, and terpene acids), and heavy metal ions (iron, nickel, aluminium, chromium, etc.) [22,35,36,37,38]. Figure 1 shows the main inhibitors in lignocellulosic materials. With increased knowledge and understanding of the mechanisms of inhibition and detoxification, it is understood that specific chemical functional groups are responsible for the inhibitory effect and toxicity to microbes [11,59]. Naming the inhibitors by functional group implies likely mechanisms of the inhibition, and potentially helps to facilitate the investigation and understanding of the detoxification of the inhibitors; therefore, the inhibitors are classified into different groups and related to the origin in the plant cell.

Due to the heterogeneous nature of lignocellulosic biomass, the degradation of byproducts produced during the fermentation can vary significantly. The variety and concentration of inhibitory compounds also depend upon the raw material used, the pretreatment conditions such as treatment materials, temperature, pH, pressure, and time duration, and the amount of solids in the reactor [35,36,37,42,59,61,62,63]. In general, it was observed that low-molecular-weight compounds show more toxic effects to microbes than do high molecular-weight compounds [64]. This property could perhaps be ascribed to an easier transport of the smaller molecules via a variety of mechanisms, including passive diffusion [59]. Enzymatic hydrolysis of lignocellulosic biomass may also release inhibitors from biomass. Organic acids such as ferulic and *p*-coumaric acids can be obtained during fermentation and saccharification from the arabinoxylan. These acids from the biomass structure are toxic to fermentation microorganisms [51].

### 2.1. Furan Inhibitors

Pentoses and some hexoses are released from hemicelluloses, from which 2-furaldehyde (furfural) and 5-hydroxymethyl-2-furaldehyde (5-hydroxymethylfurfural; HMF) can be formed by dehydration of these sugars at high temperature and acidic conditions [11,59,65,66]. Furfurals are generated during xylose degradation while HMF is generated during hexose degradation [22]. Furfural and HMF are furan derivatives and commonly called ‘furan inhibitors’. Evidence has shown that the metabolic conversion products of furfural and HMF, furan methanol and furan-2,5-dimethanol, are also furan derivatives, but less toxic to fermentative microorganisms [48,59,67].

Furan inhibitors are considered particularly undesirable due to their relative abundance and toxic effect [41,68,69,70]. The inhibitor and toxic effects appear to be caused by the aldehyde functional group rather than the furan ring [59]. Furfural and HMF are usually the representative inhibitors of yeast and bacterial growth and fermentation [11,36,42,71,72,73]. In addition, they inhibit the glycolytic enzymes used to liberate the sugars from the (hemi-)cellulose fractions and interfere with the activity of dehydrogenases, causing a reduction in growth rates and cell yields [2,11,49,74,75]. HMF, a toxic compound originating from the degradation of hexose, is the most important intermediate product in the acidic hydrolysis process. Its inhibitory effect is similar to that of furfural, causing a longer lag phase during growth [36]. However, HMF is considered less toxic than furfural [76,77].

Furan inhibitors can also be used as byproducts. Furfural is chemically produced at large scale for application as a solvent or as a building block for resins. HMF and furfural are additionally applied as flavour compounds and in the manufacture of pharmaceuticals [2,78,79,80].

### 2.2. Weak Acids

HMF and furfural can further break down to produce levulinic acid, formic acid, and furonic acid when the severity factor increases [43]. In the case of acetic acid, it is formed from the acetyl group of hemicellulose fraction [43,59].

The toxicity of these acids is mainly due to its undissociated form; thus, the medium pH is important [37,54,77]. Acids disrupt cellular energy generation by collapsing pH gradients especially at low pH [45]. Although formic acid has a low pKa of 3.75 and thus a lower concentration of undissociated molecules at the pH prevailing in fermentation, it is more toxic to the yeast due to its small size compared with acetic acid (pKa 4.75) and levulinic acid (pKa 4.66). The small size of the formic acid molecule is thought to increase its mass transport through the cell wall; after entering the cytosol, the acid dissociates, lowering the pH and inhibiting cell growth. The organic acids inhibit the yeast when the concentration is so high that yeast cells start to die and they also partially deactivate enzymes [11,65,81,82]. The relative toxicity is a function of hydrophobicity because this characteristic determines the ability of the compound to pass through the membrane [45,49]. Alcohols are generally less toxic than related acids or aldehydes, but their toxicity is also related to hydrophobicity. They appear to cause a breakdown in membrane structure [46].

### 2.3. Phenolic Compounds

Phenolics, another inhibitor generated from lignin breakdown, may exist in three different forms: acid, ketone, and aldehyde (e.g., catechol, vanillic acid, syringic acid, vanillone, syringaldehyde, and conferyl aldehyde). Among other inhibitory derivatives of phenolics, 4-hydroxy benzoic acid, ferulic acid, and guaiacol are the most commonly observed in lignocellulose acid hydrolysates [11,22,36,42,59]. Phenolics have been reported to be among the most toxic compounds to fermentation microorganisms [42,51,54,65]. The phenolic compounds are toxic to the yeast; phenolics partition into membranes and lead to loss of integrity, interfering with cell growth and sugar transport [49,83].

In the same way as other inhibitors, phenolics can be used for several applications. Some of them are based on their antioxidant activity against reactive species involved in aging and in chronic, autoimmune, inflammatory, coronary, and degenerative diseases [84,85,86,87]. Their antioxidant properties may explain a part of the potential cancer chemopreventive properties [88], although the antioxidant activity alone is not sufficient to explain their whole set of biological properties [89,90,91,92].

### 2.4. Other Inhibitors

The lignocellulosic raw material also generates tannic acid, terpenes, and other polymers upon chemical degradation. In addition, SO_2_ from the raw material hydrolysis may inhibit fermentative reactions, being harmful to microbial growth and metabolic activities [22,37,59]. Heavy metal ions (iron, chromium, nickel, and copper) can originate from corrosion of hydrolysis equipment. Although they are not always produced in large quantities, they can have some toxic effect on the alcoholic fermentation microorganisms [37,51].

### 2.5. Synergistic Effects

The sum of the effects of all toxic compounds in hydrolysates is almost certain to be more than the sum of the parts. Synergies have already been detected in simple combinations, and the ability to test for toxic effects on a high throughput manner will allow for the identification of more complex combinations of individual compounds or fractions. The existence of these synergies implies that alteration of pretreatment and conditioning steps to eliminate a single member of a synergistic combination could have a greater impact than elimination of compounds acting alone. It also helps explain why enhanced resistance to furfural alone can improve fermentation in hydrolysate [48,49,72,93].

Synergistic and antagonistic effects are thought to occur when combinations are more inhibitory than the sum of the individual effects. Many references point out the synergistic effect of different inhibitors in lignocellulosic fermentation [22,41,44,45,47,48,49,94]. Furan inhibitors in combination with acids, especially acetic acid, have been demonstrated to have synergistic effects [44,47,48,49]. However, some combinations were less than the sum of individual components, indicating an antagonistic effect and probably due to the fact that one compound could interfere with the toxic action of the other. Examples of these protective interactions are vanillyl alcohol with catechol, coniferyl alcohol, guaiacol, hydroquinone, and methylcatechol as well as the combination of furfural with methylcatechol [49]. However, due to a lack of understanding about the synergistic interactions among inhibitors and the mechanisms of these interactions, highly inhibitor-resistant microorganisms might not be expected in the short term [51].

## 3. Physico-Chemical Detoxification Processes

The low-concentration of fermentable sugar in original samples derived from lignocellulose hydrolysates would lead to an extremely low product concentration in the fermentation process. Therefore, the removal of inhibitors and concentration of sugars in lignocellulosic hydrolysates before fermentation is becoming more and more important [18].

The formation of inhibitors during biomass (pre-)treatment may be prevented by careful control of the process parameters. Although considerable progress has been made in lab-scale hydrolysis processes [95], it should be noted that the formation of inhibitory byproducts is not easily prevented in an economical way at an industrial scale. Hence, it is often preferred to remove inhibitors prior to fermentation. Therefore, in order to facilitate fermentation processes, additional remediation treatments—including physical, chemical, or biochemical detoxification procedures—are often required to remove these inhibitory compounds [37,59,96]. Several techniques have been proposed for the hydrolysate detoxification, including overliming or neutralisation [35,55,65,96,97], adsorption [10,37,98,99,100,101,102,103,104,105,106,107], liquid–liquid extraction [63,108], evaporation [62,65,109,110], and enzyme or microorganism treatment [57,65,111,112].

Detoxification methods can be divided into the following three main groups [3]: biological, physical, and chemical. Biological treatments involve the use of microorganisms or enzymes that act on the toxic compounds present in the hydrolysate by changing their chemical structures [57,111]. The physical methods promote the removal of toxic compounds from the medium without changing their chemical structures [10,62,63,102,105]. On the other hand, the main chemical detoxification methods employed in hydrolysate treatment are based on the addition of reductive substances and pH modification [35,96,113].

Nevertheless, the effectiveness of a detoxification method depends on (i) the type of hemicellulosic hydrolysate, because each type of hydrolysate has a different degree of toxicity; (ii) the concentration of inhibitors; and (iii) the microorganism being used, because each species of micro-organism has a different degree of tolerance to inhibitors [50,53,54,59,114,115]. Furthermore, as each detoxification method is more specific for certain types of compounds, better results could be obtained by combining two or more different methods [50].

On the other hand, inhibitor removal is a very selective process and it is difficult to identify a standard process that provides satisfactory results for all substrates. In addition, not all potentially inhibitory compounds have been identified to date. It is possible that some undiscovered compounds have synergistic inhibitory effects even at low concentrations, as is the case for the aldehyde inhibitors furfural and HMF. Therefore, continuing efforts to identify and understand the profiles of inhibitory compounds present in various hydrolysates remains a critical area of research for enabling the development of improved detoxification methods. Considering the need to keep low the process costs of commodity products such as ethanol, the removal of inhibitors from hydrolysates using the abovementioned methods may not be an economically worthwhile approach given the costs associated with additional processing steps and the loss of fermentable sugars [59].

However, these additional steps add cost and complexity to the process and generate extra waste products. Economic improvements in biofuel and bioproduct production could be achieved if these inhibitors could be eliminated from the hydrolysates, as they limit their efficient utilisation for value-added products of commercial interest [22].

The physico-chemical detoxification processes for lignocellulosic materials have been evaluated in this review. Results from the literature have been graphed and discussed in relation to the main inhibitory compounds and taking into account the lignocellulosic raw material.

### 3.1. Evaporation

**Figure 2:**
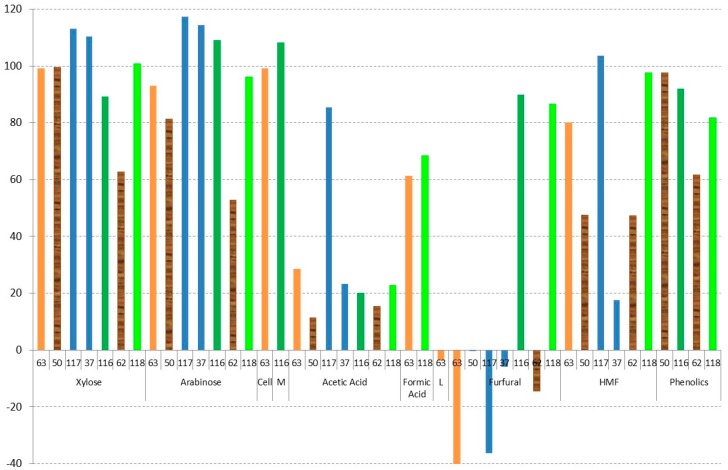
Results of sugar and inhibitors concentration during vacuum evaporation for 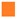 corn stover [63], 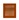 eucalyptus wood [50,62], 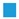 rice straw [37,117], 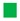 soybean hulls [116], and 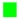 olive tree pruning residue [118] hydrolysates. The number included in the *x* axis is related to the reference number. Cell: Cellobiose, M: Mannose, L: Levulinic acid.

Vacuum evaporation is a physical method that is used to reduce the amounts of volatile compounds present in different hydrolysates; therefore, it is considered a detoxification procedure [50,59]. Figure 2 shows the results of vacuum evaporation by different authors [37,50,62,63,116,117,118]. A different colour in columns has been used in relation to the lignocellulosic raw material. In addition, in order to compare the obtained results, all of the data have been correlated to the concentration factor employed based on glucose (100% being the same concentration factor as glucose). In all cases, 70 °C has been used in the vacuum evaporation process. As can be observed in Figure 2, all sugar content has similar results to glucose with concentration factors between 89% and 117% [37,63,116,117,118], giving the same concentration in the evaporation unit, except for xylose and arabinose in the case of 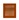 eucalyptus wood hydrolysates, whereas xylose and arabinose are between 51% and 62% in the case of eucalyptus hemicellulosic hydrolysates [62] and from 81% to 99% in the case of *Eucalyptus grandis* [50], pointing out the importance of the optimisation of this method for hydrolysates with more pentose sugar content. On the other hand, when vacuum evaporation for 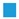 rice straw hydrolysates is used [37,117], a slight increase of the xylose (13% higher) and arabinose (15%–17% higher) is found in relation to glucose.

Evaporation can be used to detoxify hemicellulosic hydrolysates in the case of acids [37,50,62,63,116,117,118] and furans inhibitors [37,50,62,63,116,117,118]. Close to 80% of the acetic acid in relation to glucose is evaporated at 70 °C [37,50,62,63,116,118]. Huang et al. [117], however, only recovered a small fraction of acetic acid in rice straw, in this case, because a previous overliming process was used in the hydrolysate. In the case of furan derivatives, however, the results are more dispersed. Very good results of evaporation of furfural are obtained in all cases except for 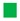 soybean hulls hydrolysate [116] and 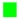 olive tree pruning hydrolysates [118]. In both cases, the reason was probably due to the pH of the sample, close to 5.5. Therefore, a previous neutralisation of the liquor is not recommended to remove this kind of pollutant; however, if the valorisation of this compound is the objective, a previous neutralisation is recommended. Regarding HMF, worse results are obtained in all cases. The best result in this case was in the sample of 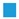 rice straw hydrolysate with no previous neutralisation, giving a detoxification of more than 80% in relation to the final concentration of glucose [37]. Regarding the concentration of phenolics, a final percentage between 62% and 92% in relation to the concentration of glucose is given; therefore, only a maximum evaporation of about 40% is obtained.

### 3.2. Liming and Overliming

Several chemical methods have been applied to precipitate toxic compounds such as alkali treatment using Ca(OH)_2_ or NaOH. By employing this overliming treatment, the pH of the hydrolysate can be increased to 9–10, and subsequently readjusted to an appropriate value using acid addition prior to microbial fermentation. This method in general reduces aldehyde and ketone inhibitors, including furfural and HMF, and improves microbial growth and fermentation performance [35,36,96].

Treatment of the hydrolysate with Ca(OH)_2_ prior to fermentation, referred to as overliming, is one of the most efficient detoxification methods and has been commonly used in studies reported previously. However, one drawback with overliming is the formation of a calcium sulphate precipitate. Another limitation is a considerable degradation of fermentable sugars if it is done under too harsh conditions (high pH and high temperature). In addition, a very harsh overliming condition might cause quantitative degradation of some inhibitors. Thus, the detoxifying treatment must be systematically evaluated to determine the optimum conditions where a high improvement in fermentability is achieved with the lowest sugar degradation [77].

**Figure 3:**
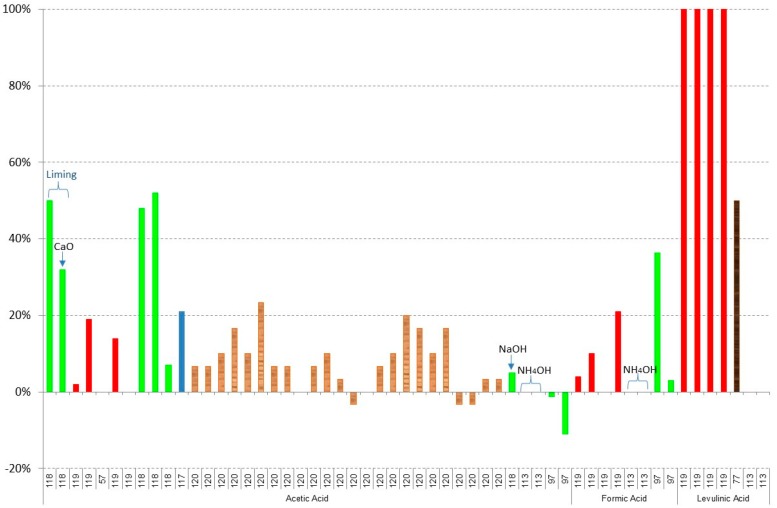
Results of acid removal during liming and/or overliming for 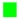 olive residues [97,118], 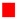 brewery’s spent grain [119], 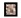 sugarcane bagasse [57,113], 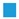 rice straw [117], 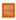 spruce [113,120], 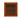 and *Kappaphycus alvarezii* [77] hydrolysates. The number included in the *x* axis is related to the reference number.

**Figure 4:**
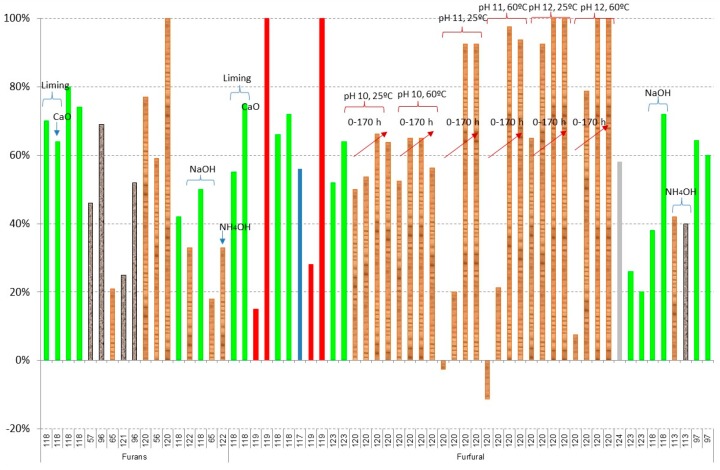
Results of furans and furfural removal during liming and/or overliming for 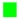 olive residues [97,118,123], 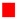 brewery’s spent grain [119], 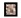 sugarcane bagasse [57,96,113,121], 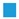 rice straw [117], 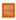 spruce [56,65,113,120,122], and 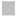 synthetic [124] hydrolysates. The number included in the *x* axis is related to the reference number.

**Figure 5:**
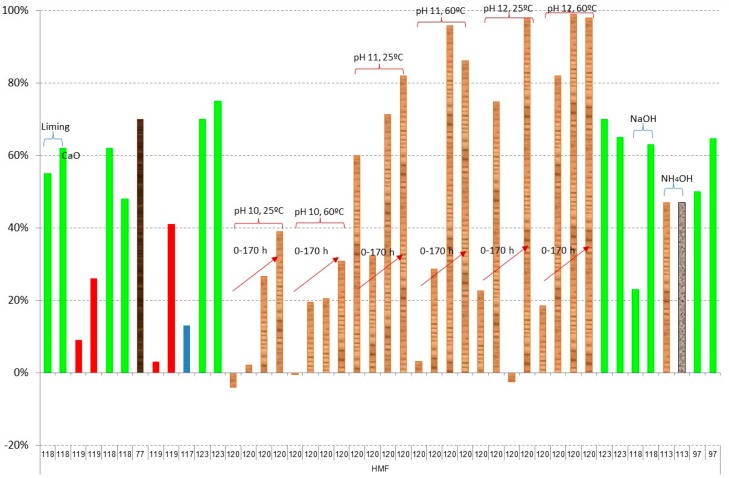
Results of HMF removal during liming and/or overliming for 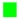 olive residues [97,118,123], 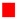 brewery’s spent grain [119], 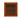 *Kappaphycus alvarezii* [77], 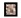 sugarcane bagasse [113], 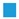 rice straw [117], and 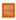 spruce [120] hydrolysates. The number included in the *x* axis is related to the reference number.

**Figure 6:**
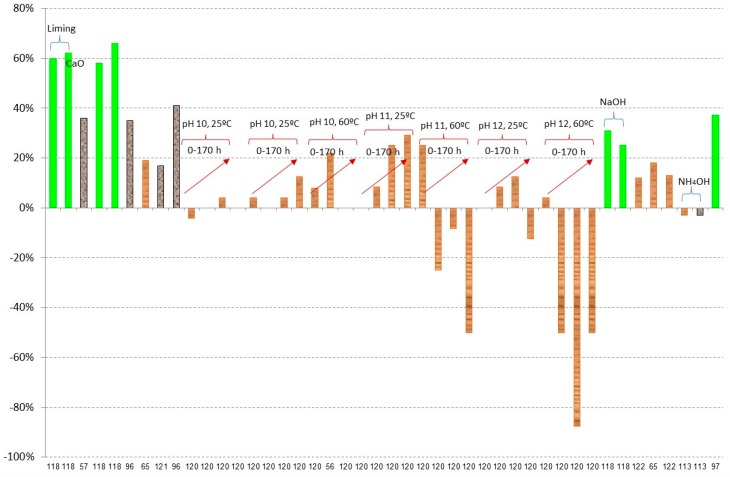
Results of phenolics removal during liming and/or overliming for 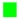 olive residues [97,118], 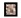 sugarcane bagasse [57,96,113,121], and 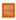 spruce [56,65,113,120,122] hydrolysates. The number included in the *x* axis is related to the reference number.

**Figure 7:**
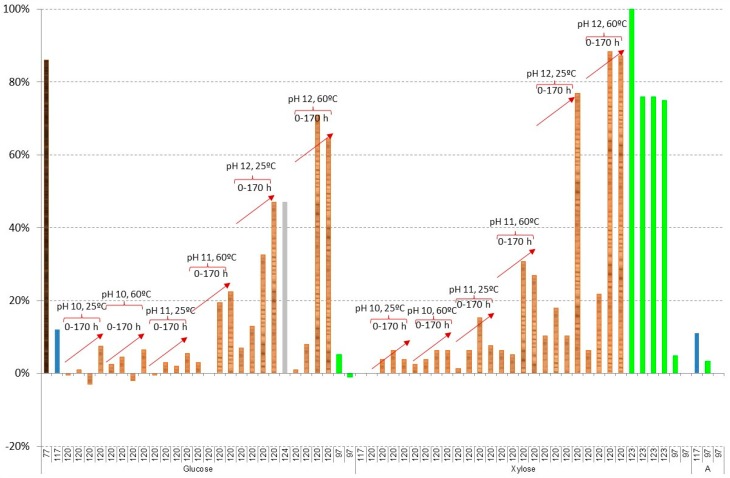
Results of losses of glucose, xylose, and arabinose during liming and/or overliming for 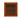 *Kappaphycus alvarezii* [77], 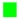 olive residues [97,123], 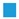 rice straw [117], 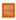 spruce [120], and 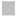 synthetic [124] hydrolysates. The number included in the *x* axis is related to the reference number. A: Arabinose.

**Figure 8:**
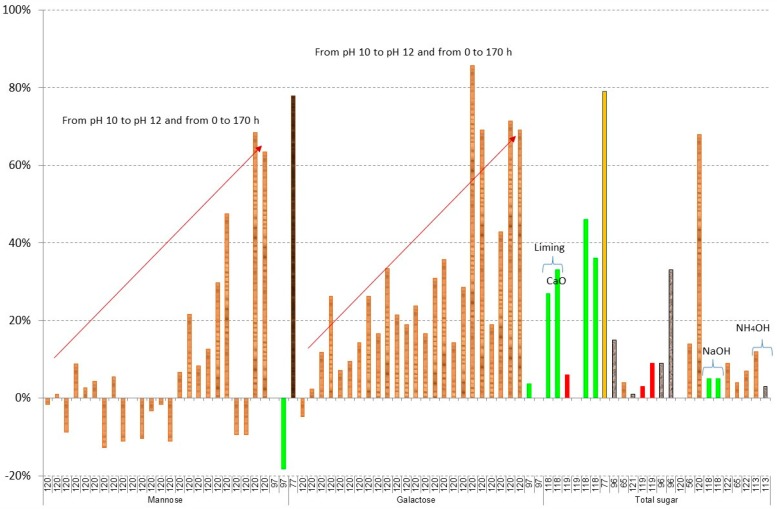
Results of losses of mannose, galactose, and total sugar during liming and/or overliming for 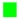 olive residues [97,118], 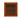 *Kappaphycus alvarezii* [77], 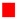 brewery’s spent grain [119], 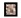 sugarcane bagasse [96,113,121], and 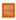 spruce [56,65,113,120,122] hydrolysates. The number included in the *x* axis is related to the reference number.

Figure 3, Figure 4, Figure 5, Figure 6, Figure 7 and Figure 8 show the obtained results of overliming or liming from the literature [56,57,65,77,96,97,113,117,118,119,120,121,122,123,124]. In all cases, the lignocellulosic raw material used in the papers is shown by a different colour. Figure 3 shows the results of weak acid (acetic, formic, and levulinic acids) removal. Negative values are due to the dilution or concentration of the sample during the experiment and the negative value of removal means that the final concentration of the inhibitor is higher after the treatment. In all cases, no big differences among the raw material are detected with slightly higher values of removal when 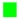 olive residues have been detoxified. The best results have been given in the case of levulinic acid for 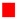 brewery’s spent grain hydrolysates [119]. In this case, Ca(OH)_2_ at pH 10 and 1 h of process is used. Results close to 50% of acetic acid have been obtained in the case of 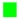 olive residues liming at pH equal to 5.5 using Ca(OH)_2_ during 10 min. The same results have been obtained when overliming at pH 10 with Ca(OH)_2_ or CaO during 10 min followed by a decrease of the pH to 5.5 with H_2_SO_4_ is used [118] and for formic acid when overliming with Ca(OH)_2_ is used with a previous water extraction [97]. The detoxification of weak acids in the rest of the experiments is close to 20%.

Figure 4 and Figure 5 show the results of furan derivatives. In all cases, a great variability of results is obtained, depending on the experiments; however, two different behaviours can be observed in relation to the raw material: (i) in the case of using 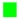 olive tree pruning or olive stones, 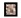 sugarcane bagasse, 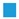 rice straw, and 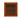 *Kappaphycus alvarezii* (cottonii), a maximum of 80% detoxification is obtained; however; (ii) when 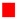 brewery’s spent grain hydrolysate or 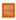 spruce hydrolysate are treated, close to 100% is obtained in both furfural and HMF in most cases [119,120]. In all cases, an increase in time (red arrows in the figure) and pH in the experiments gives better results of both furfural and HMF; however, the increase of temperature does not affect the detoxification process as much. In the results of Millati et al. [120], the use of Ca(OH)_2_ with a pH close to 12 with a reaction time of more than 20 h is recommended to obtain detoxification results close to 100%. When NaOH or NH_4_OH is used, instead of Ca(OH)_2_, maximum percentages of removal between 33% and 43% in the case of furfural and 23% and 47% for HMF are obtained, with the best results, from 40% to 47%, occurring when NH_4_OH is used [113,118].

Figure 6 shows the results of phenolics. In this case, the treated lignocellulosic material has a great influence on the final results, giving maximum detoxification results of 66% when 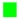 olive tree residues are treated [118], 41% for 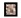 sugarcane bagasse [96], and 29% in the case of 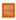 spruce hydrolysates [120].

Figure 7 and Figure 8 show the results of losses of sugars during liming or overliming. In the case of glucose and xylose, good results are obtained in most cases, with the exception of using 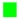 olive stones as raw material with losses from 76% to 100% of xylose [123], hydrolysate of 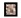 *Kappaphycus alvarezii* with losses of 86% of glucose and 77% of galactose [77], and in the most aggressive conditions, in the case of 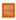 spruce hydrolysate, using a pH value of 12 with Ca(OH)_2_ and a reaction time of more than 20 h (the same conditions when furans are completely removed). In this case, losses of glucose from 65% to 71% at 60 °C and from 33% to 47% at 25 °C; xylose from 87% to 88% at 60 °C and from 75% to 77% at 25 °C; mannose from 64% to 69% at 60 °C and from 30% to 48% at 25 °C; and galactose from 69% to 71% at 60 °C and from 69% to 86% at 25 °C are obtained [120].

According to all results, liming or overliming can be used to remove some acids such as levulinic or formic acids, furans and phenolics; however, overliming does not remove acetic acids, which are known to inhibit ethanol production at concentrations greater than 2 g/L [51,125]. An optimisation of the pH between 10 and 11 and reaction time should be done, depending on the inhibitor and the raw material. Furthermore, in all cases, Ca(OH)_2_ is recommended.

### 3.3. Adsorption

Adsorption enables the separation of selected compounds from dilute solutions. Compared to alternative technologies, adsorption is attractive for its relative simplicity of design, operation and scale-up, high capacity and favourable rate, insensitivity to toxic substances, ease of regeneration, and low cost. Additionally, it avoids using toxic solvents and minimises degradation [92]. Adsorption is a technique that is used frequently in biorefineries for product polishing and removal of minor impurities [126].

Activated charcoal is the most employed adsorbent [10,57,62,77,106,116,118,119]. However, other adsorption methods for detoxification include the use of zeolite [127], eartomaceous earth [128], wood charcoal [129], diatomacenous earth [128], or polymeric adsorbents [100]. Zeolites are widely used as ion-exchange beds in domestic and commercial water purification, softening, and other applications. Zeolites have a porous structure that can accommodate a wide variety of cations, such as Na^+^, K^+^, Ca^2+^, Mg^2+^, and others, which are loosely held and can readily be exchanged in a contact solution. Eken-Saraçoglu and Arslan [127] conducted detoxification tests with CaO and combinations with zeolite during ethanol production from corn cob hemicellulose hydrolysate by *Pichia stipitis* and *Candida shehatae*. They found that the single neutralisation method did not support high ethanol production (2.8 g/L) during fermentation of hydrolysates by *C. shehatae* with only 2.8 g/L ethanol obtained. However, neutralisation and zeolite treatments significantly increased the final ethanol concentration to approximately 6.0 g/L. Wood charcoals were also tested for removal of inhibitors such as furan and phenolic compounds in wood hydrolysates [129]. Wood charcoals prepared at various temperatures were found to selectively remove only the inhibitors without reducing the levels of fermentable sugars. A wood charcoal treatment with a wood charcoal weight to hydrolysates ratio of 0.07 could enhance the fermentation of wood hydrolysates [129]. Polymeric adsorbents can also be used to remove aldehydes, such as furfural, that inhibit fermentation. Weil et al. [100] investigated the removal of furfural from a biomass hydrolysate using XAD-4 (polystyrene-divinylbenzene copolymer bead) and XAD-7 (methacrylic ester bead) polymeric adsorbents and manufactured by Rohm and Haas (Philadelphia, PA, USA). The XAD-4 showed higher specificity for furfural removal than XAD-7, and it also had little interaction with glucose.

**Figure 9:**
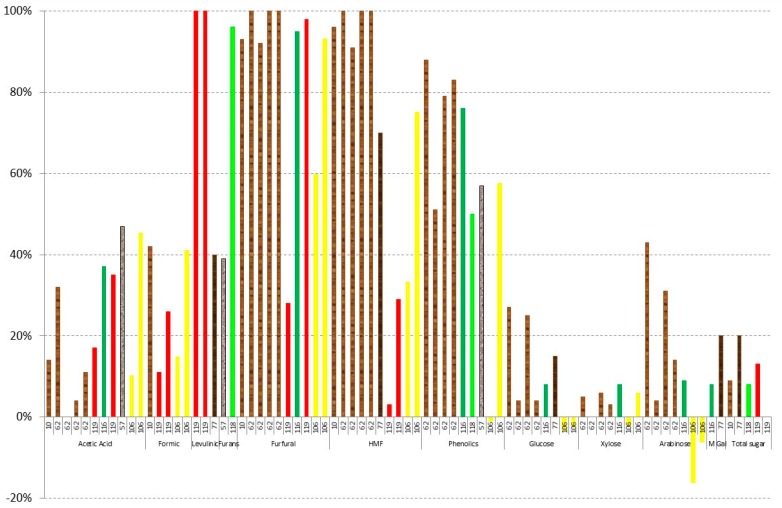
Results of losses of sugar and removal of inhibitors during adsorption with activated charcoal for 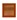 hardwood [10,62], 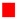 brewery’s spent grain [119], 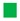 soybean hulls [116], 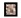 sugarcane bagasse [57], 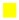 Rape straw [106], 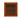 *Kappaphycus alvarezii* [77], and 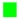 olive residues [118] hydrolysates. The number included in the *x* axis is related to the reference number. M: Mannose, Gal: Galactose.

Different authors have studied the detoxification of lignocellulosic hydrolysates by adsorption with activated charcoal. Figure 9 shows the obtained results for 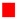 brewery’s spent grain [119], 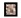 sugarcane bagasse [57], 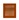 hardwood chips [10], 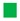 soybean hulls [116], 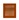 *Eucalyptus grandis* [62], 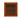 *Kappaphycus alvarezii* [77], 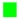 olive tree pruning residue [118], and 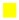 rape straw [106] hydrolysates. However, the kind of raw material has no influence on the adsorption results.

As can be observed in Figure 9, the best results are obtained for levulinic acid (from 40% to 100%), furans, furfural, and HMF (from 28% to 100%), following by phenolics (from 50% to 88%). When acetic and formic acids are removed, the highest value of detoxification is 47% for 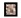 sugarcane bagasse [57] and 42% for 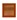 hardwood chips [10], respectively; and the losses of sugars are under 27% of glucose and 43% of arabinose in the case of 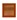 *Eucalyptus grandis* [62], 8% for mannose for 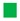 soybean hulls [116], 20% of galactose when 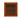 *Kappaphycus alvarezii* hydrolysates are detoxified [77], and only 8% of xylose in the case of 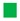 soybean hulls [116]. On the other hand, regarding the adsorption of acetic acid, in spite of having a low value, the best results are obtained in the case of using lower pHs in the hydrolysate, from 1.8 to 2.5, according to the results of Villareal et al. [62] and Schirmer-Michel et al. [116]. This behaviour is also shown in the results of HMF and phenolics; however, the losses of sugars in this case are higher [62].

In conclusion, adsorption treatment is recommended to detoxify different kinds of lignocellulosic materials, from hardwood to softwood and other lignocellulosic residues, giving very good results in the case of furans and phenolics and lower losses of sugars; however, some acids such as acetic and formic are not removed from the sample. For all adsorption-based detoxification methods, the reuse or recovery of the adsorbate will determine the economics and viability of the process [51].

### 3.4. Ion Exchange Resins

**Figure 10:**
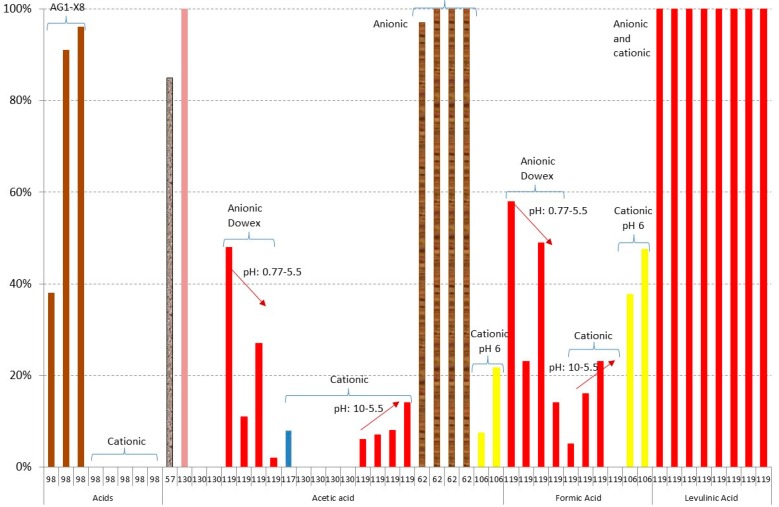
Results of removal of acids during ion exchange resin treatment for 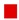 *Picea abies* [98], 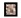 sugarcane bagasse [57,101], 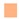 corn stover [130], 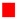 brewery’s spent grain [119], 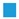 rice straw [117], 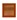 *Eucalyptus grandis* [62], 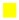 and rape straw [106] hydrolysates. The number included in the *x* axis is related to the reference number.

**Figure 11:**
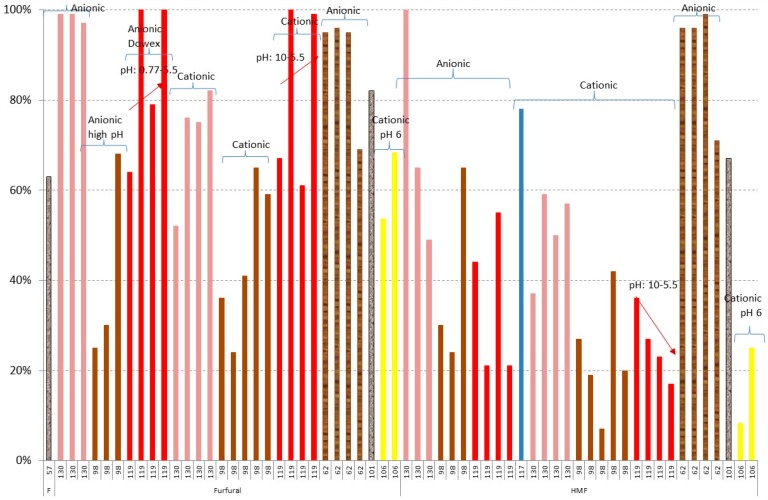
Results of removal of furans during ion exchange resin treatment for 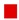 *Picea abies* [98], 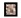 sugarcane bagasse [57,101], 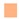 corn stover [130], 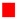 brewery’s spent grain [119], 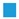 rice straw [117], 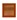 *Eucalyptus grandis* [62], and 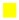 rape straw [106] hydrolysates. The number included in the *x* axis is related to the reference number. F: Furans.

**Figure 12:**
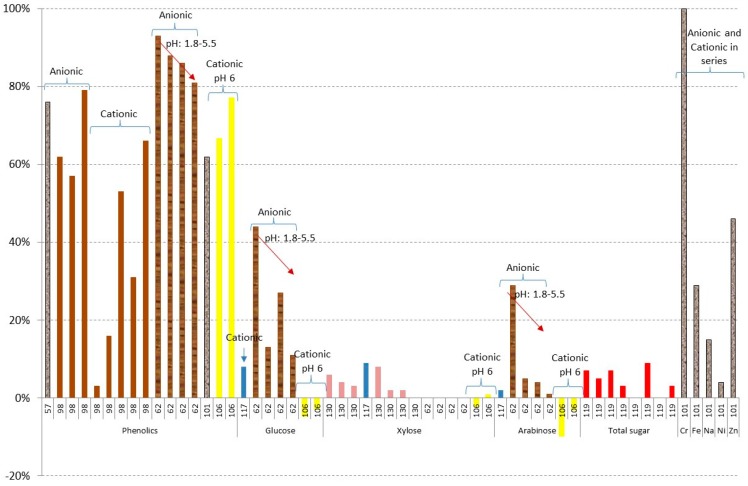
Results of removal of phenolics and heavy metals and losses of sugar during ion exchange resin treatment for 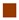 *Picea abies* [98], 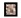 sugarcane bagasse [57,101], 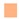 corn stover [130], 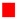 brewery’s spent grain [119], 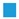 rice straw [117], 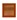 *Eucalyptus grandis* [62], and 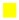 rape straw [106] hydrolysates. The number included in the *x* axis is related to the reference number.

Ion exchange resin treatment is one of the most efficient methods for lignocellulosic hydrolysate detoxification [54]. In this case, depending on the kind of inhibitor, anionic or cationic resin can be used. However, due to the complex structure of the lignocellulosic materials—all of the inhibitors are usually associated to complex molecules with anions and cations—both kinds of resins are recommended. Figure 10, Figure 11 and Figure 12 show the results of removal of inhibitors and the losses of sugars in the case of using ion exchange resins [57,62,98,101,117,119,130]. Figure 10 shows the results of detoxification of weak acids.

The best results have been obtained for levulinic acid. Regarding the rest of the acids, the best results have been obtained using anionic resins AG1-X8 (BioRad Laboratories, Richmond, CA, USA) [98] and A193 S [130] for 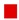 *Picea abies* and 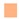 corn stover hydrolysates, respectively. In addition, very good results have been obtained for acetic acid for 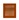 *Eucalyptus grandis* [62]. However, low results of acids (acetic and formic) are obtained for 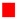 brewery’s spent grain hydrolysate [119]. When cationic resins IRN-77 and XAD-X8 (BioRad Laboratories) are used, removals up to only 14% for acetic acid and 23% of formic acid are obtained, while removals close to 100% are obtained in the case of levulinic acid [98,119]. The pH needs to be optimised in all cases, giving better results at lower pHs.

The results of furans, Figure 11, are much better in all cases, using both anionic and/or cationic resins. In this case, the most important variable is the pH value. When anionic resin is used, a pH value of 0.77 to 5.5 is recommended according to the results of Carvalheiro et al. [119] and Villarreal et al. [62]. However, in the case of using a cationic resin, an initial pH value of 10 is recommended [98]. The results of removal of furfural are higher than HMF and with respect to the raw material, the best results are obtained for 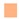 corn stover [130], 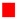 brewery’s spent grain [119], and 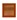 *Eucalyptus grandis* [62] hydrolysates.

Figure 12 shows the results of phenolics, metals, and losses of sugars in different ion exchange resin treatments. Phenolics are well removed in the case of using anionic resins with results from 57% to 79% [57,98,106] and only a small influence is observed for the initial pH and the raw material. In the case of metals, very good results are obtained for chromium, following by Zn (46%), Fe (29%), Na (15%), and Ni (4%) [101]. Regarding sugars, this is one of the best methods with only a small amount of sugar lost in all of the experiments. The highest losses of sugars have been obtained for 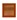 *Eucalyptus grandis* at lower pHs (1.8), giving 44% losses of glucose and 29% of arabinose [62].

### 3.5. Liquid–Liquid Extraction

In biorefineries, liquid-liquid extraction is widely implemented for recovering fuels and chemicals from biological mixtures such as fermentation broths [126]. A solvent (extractant) that is immiscible with the process solution is used to extract the solute. After extraction, the extract (extracted solute + extractant) is separated from the raffinate (original solution depleted of the solute) by another unit operation, most commonly a gravity settler. The solute is recovered from the extract by evaporating the extractant. Extraction is an equilibrium-governed process that relies on the distribution of the solute between the original and extracting solvents. Important factors for selecting the extraction solvent include: partition coefficient (distribution constant), immiscibility with the original solvent, and boiling point for evaporation [126].

**Figure 13:**
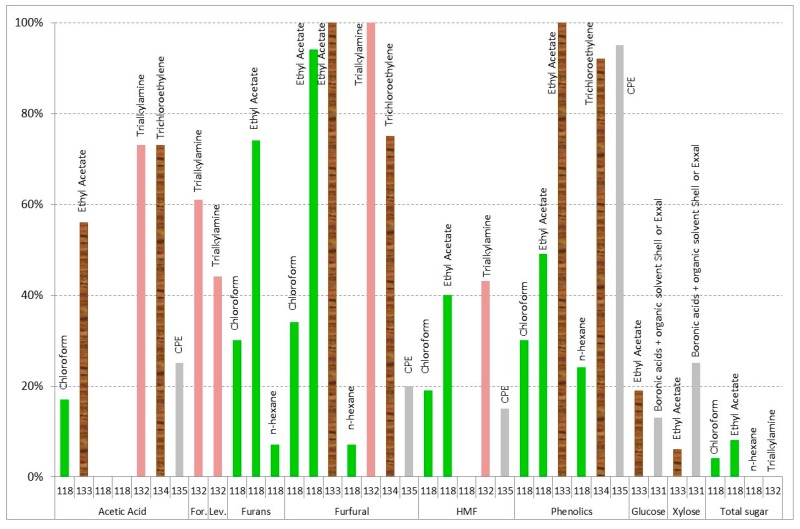
Results of losses of sugar and removal of inhibitors during solvent extraction for 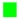 olive residues [118], 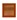 hardwood [133,134], 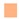 corn stover [132], and 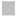 synthetic [131,135] hydrolysates. The number included in the *x* axis is related to the reference number. For: Formic acid, Lev: Levulinic acid.

Figure 13 shows the obtained results of solvent extraction. Chloroform, ethyl acetate, *n*-hexane, trialkylamine, trichloroethylene, cloud point extraction (CPE), and boronic acids with organic solvent have been used for 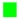 olive tree pruning residue [118], sugarcane bagasse [131] 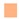 corn stover [63,132], aspen [133], 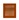 wood [134], and 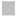 synthetic [135] hydrolysates. Both ethyl acetate and trialkylamine give the best results for furans and phenolics [63,118,132,133,134], trialkylamine, and trichloroethylene in the case of acids [63,132,134]. Wilson et al. [133] found that ethyl acetate extraction was more effective than roto-evaporation in removing the inhibitors. The roto-evaporation removed furfural and most of the acetic acid but did not reduce lignin-derivative levels. The ethyl acetate extraction removed all the inhibitory compounds, except acetic acid, which was not completely removed by the ethyl acetate extraction process [51,133].

Cloud point extraction can be used for phenolics [135]. The surfactant-based cloud point extraction aqueous two phase system is a new method having the potential for separation and recovery of inhibitors. Cloud point extraction is an upcoming technology to preconcentrate and separate many of the trace elements from different chemical and biological systems. The system is sustainable as it involves benign extractants like surfactants and low concentrations at slightly elevated temperatures to form clouds that separate out from the bulk solution [136].

### 3.6. Filtration by Membrane Operations

Membrane technologies, especially the pressure-driven membrane filtration, are efficient, cost-competitive, and promising separation methods during industrial production process [137]. In integrated biorefineries, membrane-based separation technologies are becoming more widely deployed due to their versatility, separation efficiency, energy savings, and economic benefits [126,138]. They are used in the food, pharmaceutical, biotechnological, bioprocessing, and chemical industries. A membrane is a porous, semi-permeable separation medium that fractionates different species from a solution based on size, shape, solubility, or molecular interactions. The permeate solution containing the “smaller” species penetrates through the membrane, whereas the retentate solution containing the “larger” species is rejected by the membrane. Membranes are fabricated from many materials including inorganics such as alumina or silica or organics such as polyethersulfone, polyamides, or cellulose acetate. Membranes are commercially available in different module formats, including tubular, hollow fibre, flat sheet, spiral wound, etc. Membranes can be fabricated with pore diameters ranging from <1 nm (virtually non-porous) to 10 µm.

Applications of membrane technology for sugar fractionation, sugar concentration, and inhibitor separation from lignocellulose hydrolysates have been studied in recent years. Microfiltration, ultrafiltration and nanofiltration are the widely used membrane filtration processes in biorefineries. The pore diameters of the membranes are in the range of 2 nm to 50 nm for ultrafiltration and 50 nm to 5 µm for microfiltration [126]. Membrane operations are used especially in the case of lignin derivatives in order to separate the lignin fraction to the hemicellulosic content [139]. However, unfortunately, wood hydrolysates have a high fouling tendency that might lead to inefficient process operations due to decreased filtration capacity and increased costs. It is difficult to obtain detailed information about the main foulants because the composition of the wood hydrolysate is very complex, containing many challenging components, and studies focusing on fouling in biorefinery applications are thus far not widely available. Fouling of membranes leads to increasing costs because of a decrease in filtration capacity, an increase in the number of membrane cleanings required, and a decrease in membrane lifetime [139]. To be able to effectively separate hemicelluloses with ultra- or microfiltration, fouling problems should be prevented or at least mitigated. This could be done by pretreating the wood hydrolysate to remove possible foulants before ultrafiltration [139]. Several methods can be used to prevent fouling problems in membrane operations of lignocellulosic materials, such as liming or overliming, centrifugation, liquid–liquid extraction, or adsorption. According to Koivula et al. [139], the best results were given by the use of adsorption and/or pulse corona discharge treatments.

**Figure 14:**
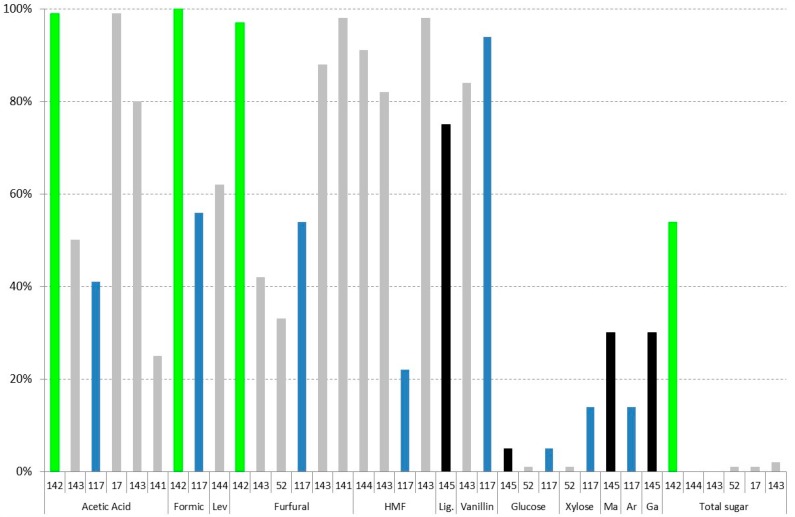
Results of losses of sugar and removal of inhibitors during membrane operations for 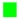 olive residues [142], 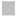 synthetic [17,52,141,143,144], 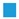 rice straw [117] hydrolysates, and 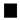 black liquor [145]. The number included in the *x* axis is related to the reference number. Lev: Levulinic acid, Lig.: lignin, Ma: Mannose, Ar: Arabinose, Ga: Galactose.

Figure 14 shows the results of filtration by membrane operations in detoxification of lignocellulosic hydrolysates. In all cases, very good results have been obtained, except for 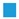 rice straw [140] and some 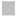 synthetic [52,141] hydrolysates. However, depending on the kind of sugar, high losses can be obtained, from glucose (up to 5%), xylose and arabinose (up to 14%), and mannose and galactose (up to 30%) [17,52,141,142,143,144,145].

### 3.7. Combination Processes

Table 1 shows a summary of all of the physico-chemical processes described in this paper. Main removal of inhibitors, conditions, advantages, and disadvantages are shown in the table. In addition, other advantages for all of them are the simplicity of design, operation, and scale-up. In some cases, the organic solvents, resins, or adsorbents can be regenerated and the separation of the inhibitors from the sugar substrate is easy, giving some other possibilities of valorisation. The costs of all of these processes are not high, depending mainly on the reagents and materials (solvents, membranes, resins, and adsorbents), with the best option being the overliming process.

**Figure 15:**
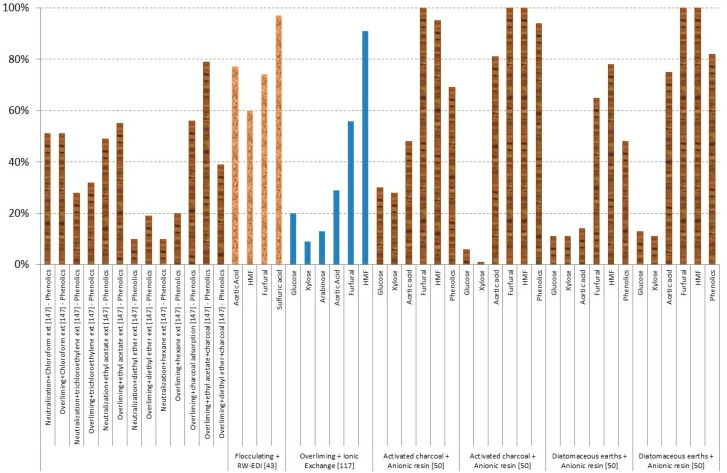
Results of losses of sugar and removal of inhibitors when a combination of processes is used for 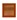 eucalyptus wood [50,147], 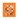 ponderosa pine wood [43], and 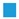 rice straw [117] hydrolysates.

However, often a combination of different inhibitor removal methods is more efficient than any single method alone to remove a variety of inhibitory compounds, such as applying pH adjustments, activated charcoal adsorption, boiling, and/or evaporation [59,146]. Figure 15 shows the obtained results in combination processes. 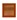 Eucalyptus wood [50,147], 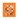 ponderosa pine wood [43], and 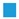 rice straw [117] hydrolysates have been studied. The best results are obtained for overliming + ethyl acetate extraction + activated charcoal adsorption for phenolics for eucalyptus wood hydrolysates [147] and the use of activated charcoal or diatomaceous earths + anionic resin in the case of furans for eucalyptus wood [50]. In addition, using flocculation + resin-wafer electrodeionisation (RW-EDI), good results in all of the inhibitors have been obtained, with a removal of 60%–74% of furans, 77% acetic acid, and 97% of sulphuric acid when Ponderosa pine wood hydrolysate is used as the raw material [43]. Both processes, flocculation + resin-wafer electrodeionisation, are explained in the following section.

### 3.8. Other Processes

#### 3.8.1. Steam Stripping

Steam stripping, also known as steam distillation, is a process of removing temperature sensitive compounds that cannot be separated by normal distillation due to decomposition at high sustained temperatures. It removes volatile inhibitors or inhibiting end-products such as furfural and acetic acid; the same as evaporation, the main disadvantage is that this process cannot remove several compounds from the lignin content [51].

#### 3.8.2. Reducing Agents

The addition of reducing agents to fermentation media improved their fermentability. Three methods have been proposed for overcoming unfavourable oxidation-reduction potential in this media: phytochemical reduction by large amounts of yeast; use of reducing agents; and production of reducing substances from sugars by either caramelisation or alkali degradation [51]. When Na_2_SO_3_, NaHSO_2_, Na_3_SO_3_·5H_2_O, Na_2_S_2_O_3_, Na_2_S_2_O_5_, KHSO_3_, Na_2_S, sulphite waste liquor, alkali-decomposed sugar, ascorbic acid, cysteine, or reduced iron filings were added to the hydrolysates, an improved fermentation was observed [51]. Diethanolamine, triethanolamine, pyridine, aniline, dimethylaniline, and similar substances also showed favourable action toward fermentation under the same conditions. The amount of reducing agent required is dependent upon the length and temperature of the heat treatment period. The mechanism of detoxification by reducing agents is not clear. However, researchers have found that toxic and oxidizing compounds such as furfural and HMF would be reduced to their less inhibitory alcohol forms inside yeast cells associated with oxidation of NAD(P)H, and redirect yeast energy to fixing the damage caused by furans and by intracellular reduced NAD(P)H and ATP levels [51,81,148].

#### 3.8.3. Other Membrane Processes

There are some other membrane-based processes, such as electrodialysis, electrodeionisation, pervaporation, vapour permeation, membrane distillation, and supported liquid membranes that are used frequently, but not included in the filtration spectrum. Among them, electrodialysis and electrodeionisation are charge-based membrane separations processes that operate under the driving force of electrochemical potential and separate charged species from uncharged species or fractionate multi-charged species. Pervaporation and vapour permeation operate under the driving force of chemical potential and fractionate organic/water mixtures with the help of a permselective (non-porous for all practical purpose) membrane. The permeate transports across the membrane in the gas phase. Membrane-based processes that are relevant in integrated biorefineries are described below [126].

Electrodialysis is an ion exchange membrane process that uses an electrical potential as a driving force. Its system typically consists of a cell arrangement with a series of alternating anion and cation exchange membranes between an anode and a cathode to form individual cells having a volume with two adjacent membranes. Electrodialysis has been widely applied to bioseparation processes to separate organic acids such as lactic acid, citric acid, acetic acid, and their salts including conventional applications to mineralise water, desalinate saline solutions, produce table salt, and treat wastewater. However, membrane fouling, which takes place due to deposition of organics on the membrane surface, is one of the most significant considerations [149]. In biorefineries, electrodialysis was used to remove acids from mixed wood hydrolysate [150], but the effect of removal on the fermentation performance was not studied in a systematic way. Rather, several batches were analysed and the fermentable sugars (glucose, galactose, mannose, and xylose) ranged from 10 to 121 g/L; acetic acid from 0.43 to 6.2 g/L; HMF from below limit of detection to 2.2 g/L. Though the different batches supported varied fermentation results with *C. shehatae* strain PFL-Y-049, it is not possible to draw conclusions regarding the efficacy of electrodialysis because no unconditioned hydrolysate batches were used as controls [49]. According to the results of Lee et al. [149], the electrodialysis process was effective for removing the fermentation inhibitors (acids, phenolics, and metals), and the fermentable sugar concentrations were unaffected. Most of the acetic acid was removed due to its ionic properties. Phenolics were removed with an efficiency of >50% under all pretreatment conditions. It is assumed that the removal of non-ionisable hydrophobic inhibitors is related to their rejection from the membrane surface, as ion exchange membrane surfaces have hydrophilic properties. However, most of the HMF and furfural, which are also non-ionisable hydrophobic inhibitors, remained in the hydrolysate after electrodialysis, showing low removal efficiency for all experiments.

Electrodeionisation is a modified version of electrodialysis that contains conductive ion exchange resin beads within the diluate compartment. Electrodeionisation combines the advantages of electrodialysis and conductive ion exchange resin chromatography. It utilises in situ regeneration of the conductive ion exchange resin beads by a phenomenon known as “water splitting”. Water splitting on the surface of the resin beads regenerates the beads and ensures higher ionic conductivity within the diluate compartment [126]. In conventional electrodeionisation, loose ionic exchange resin beads are used; however, the researchers at Argonne National Laboratory have improved the technology by using resin wafers to incorporate the loose ion exchange resin. The modified platform is called Resin-wafer electrodeionisation. Argonne patented the technology to fabricate and use the resin wafers [151]. The technology offers enhanced flow distribution, higher conductivity, superior pH control, ease of material handling and system assembly, and a porous solid support for incorporation of catalysts, biocatalysts, and other adjuvants. The pH can be electrochemically controlled, enabling selective removal of acids or other charged species based on the isoelectric point. At low conductivity, resin-wafer electrodeionisation offers a significant decrease in power consumption compared to electrodialysis. In comparison to conventional ion exchange columns, it does not have to be regenerated with stoichiometric amounts of acids/bases. Rather, in situ regeneration of the resin beads in electrodeionisation takes place by water splitting due to the applied electric field [43].

Resin-wafer electrodeionisation is one of several processes than can be used to remove organic and mineral acids from solutions, an alternative to reduce the overliming cost. Resin-wafer electrodeionisation has been used extensively for production of boiler grade water from impaired sources, high fructose corn syrup desalination, desalination of glycerol, production and recovery of organic acids [152], especially organic acids from fermentation broth [153], post-transesterification glycerine desalting [154], conditioning of biomass hydrolysate liquor [155], and for CO_2_ capture from flue gas [156]. According to the results of Lin et al. [156], using resin-wafer electrodeionisation, >99% sulphuric acid and >95% of acetic acid were removed. For the neutral xylose sugar, >98% was retained. By adjusting the operating conditions, selective separation of sulphuric acid and acetic acid was achieved to obtain two separate acid enriched streams. For a typical case, the sulphuric acid-enriched stream contained around 20 g/L of sulphuric acid and 1 g/L of acetic acid. On the other hand, the acetic acid-enriched stream contained around 0.5 g/L of sulphuric acid and 9 g/L of acetic acid. The sulphuric acid stream could be recycled back for the dilute acid pretreatment, while the acetic acid stream could be recovered as a value-added biobased co-product.

#### 3.8.4. Aqueous Two-Phase Extraction

Aqueous two-phase systems are clean alternatives for traditional solvent extraction systems. These techniques are formed when two polymers, or one polymer and one salt are mixed together at appropriate concentrations and at a particular temperature. The two phases are mostly composed of water and non-volatile polymers, thus eliminating the use of volatile organic solvents. Aqueous two-phase extraction is normally performed under mild conditions, for example, 25 °C, which do not harm or denature unstable/labile biomolecules or microorganisms. In this process, the interfacial stress (at the interface between the two layers) is lesser (400-fold less) than that in water-organic solvent systems used for solvent extraction, causing less damage to the molecules to be extracted. The separation of the phases and the partitioning of the compounds occur rapidly. The process has been tested for a number of years in biotechnological applications as a benign separation method. In addition, it has been investigated for extractive fermentation and removal inhibitors [51,157] from lignocellulosic hydrolysates during biofuel production from biomass. Major disadvantages of aqueous two-phase extraction include the relatively high cost of the polymer, the recycling of polymer(s), and poor selectivity, although specialized and efficient systems may be developed by varying factors such as temperature, degree of polymerization, and presence of certain ions [51].

#### 3.8.5. Supercritical Extraction

Any substance at a temperature and pressure above its thermodynamic critical point will become supercritical fluid, which can diffuse through solids like a gas and dissolve materials like a liquid. Additionally, close to the critical point, small changes in pressure or temperature result in large changes in density, allowing many properties to be adjusted. Supercritical fluids may be suitable as a substitute for organic solvents in a range of industrial and laboratory processes. However, the capital cost is expensive [51]. Supercritical fluid extraction of an acid hydrolysate of spruce removed a number of potentially toxic compounds by varying degree, resulting in improved fermentation yields and productivity with baker’s yeast as fermentation organism [158]. Furfural was reduced by 93%, coniferyl aldehyde by 91%, but HMF was only reduced by 10%, acetic acid by 19% and levulinic acid by 6%. Even the poorly removed compounds were identified in the extracted material, concentrated by the evaporation of CO_2_ [49].

#### 3.8.6. Advanced Oxidation Processes

A new, promising, and little studied method for the detoxification of lignocellulosic hydrolysates is the use of advanced oxidative processes. Advanced oxidative processes can be defined as those methods where hydroxyl radicals (HO·) are produced in sufficient quantities to act as the main oxidizing agent. The hydroxyl radical is a powerful oxidizing agent that is able to mineralize biorecalcitrant organic compounds or convert them into biodegradable compounds [159]. Due to its high reactivity, the hydroxyl radical must be generated in situ, which may be accomplished with a number of different processes. Hydroxyl radicals can be generated as a result of a combination of strong oxidizing agents, such as hydrogen peroxide and ozone. Ultraviolet (UV) or visible radiation and catalysts such as metal ions and semiconductors can also be used to create hydroxyl radicals [3]. Advanced oxidation processes have been studied for the remediation of lignin derivatives from pulp and paper industry wastewater. Such processes have the ability to degrade toxic and recalcitrant compounds, thereby reducing the toxicity of effluents and enhancing their susceptibility to biological agents. Therefore, it is possible to apply this kind of process as a method of reducing the toxicity of lignocellulosic hydrolysates [3].

#### 3.8.7. Polyelectrolytic Flocculation

That said, the use of polyelectrolyte flocculating agents with chemistries similar to ion exchange and hydrophobic interaction resins may yet provide a feasible detoxification method while minimizing sugar losses [60]. The polyelectrolyte may preferentially react or form a complex with non-inhibitory compounds, thus reducing the number of active sites available for removing the inhibitory compounds; we have already shown that chloride or sulphate ions interfere with the removal of acetic acid using PEI [160]. Similarly, inhibitory compounds might also interact with other species in solution, which could alter their ability to interact with the polymer [60].

Flocculation by polyelectrolytes can be an alternative method to remove inhibitory compounds either before or after the enzymatic hydrolysis. Polethyleneimine (PEI) is a soluble secondary amine cationic polymer, commonly used as a flocculating agent to precipitate cellular debris and other insoluble solids. It has been evaluated for removal of suspended solids from biomass slurries [161] and Carter et al. [60,160] studied the efficiency of PEI to remove furfural and HMF from clarified pre-enzymatic hydrolysis liquor [43].

## 4. Application to Lignocellulosic Materials Derived from SO_2_-Based Processes

The valorisation of lignosulphonate fractions and the upturn of dissolving pulp production have given an increase in the pulping and hydrolysis processes derived from SO_2_ such as sulphite pulping, sulphite pre-treatment (SPORL), and SO_2_-Ethanol-Water (SEW) processes [16,146,162,163].

The acid sulphite process is based on the extraction of cellulose by the attack under acidic conditions (pH of 1.35 ± 0.15) in the presence of excess free SO_2_ [16]. The main advantages of this process are (i) the production of a high-purity cellulose (dissolving pulp) for not only textile fibre production but also for high value-added films, plastics and coatings among others [163]; and (ii) the possibility to obtain a high separation of all of the main components: cellulose, hemicellulose, and lignin [16].

However, in addition to the acid sulphite process, other pretreatments such as SPORL or SEW can be used to obtain dissolving pulp. SPORL is reported to be the most energy-efficient pretreatment method in terms of sugar production per unit of consumed energy [164,165]. The first step consists of chipping woody biomass into large pieces of up to 38 mm length/width and a thickness of about 6 mm. Wood chips are then reacted for 10–30 min and at 160–190 °C, with a solution of 1%–8% bisulphate and 0.5%–2.2% sulphuric acid (on oven dry wood), depending on the wood type. The substrate is created by means of a disk refiner that separates the pretreated, softened chips at a fibre interface level [164,165,166]. The SO_2_-Ethanol-Water (SEW) process has the potential to replace the acid sulphite process for the production of rayon-grade pulps owing to a higher flexibility in the selection of the raw material source, substantially lower cooking times, and the near absence of sugar degradation products [163]. In this case, this novel fractionation process has the potential to replace the acid sulphite process owing to a higher flexibility in the selection of the raw material source, substantially lower cooking times, and the near absence of sugar degradation products. The SEW process gives more sugar substrate and fewer inhibitors than the magnesium-based sulphite process, especially furans and acids. However, no differences are seen in the properties of the dissolving pulps resulting from both acidic processes [163]. In addition, the SEW process only requires evaporation of ethanol and SO_2_ for recovery of the fractionation chemicals due to the absence of a base (Mg or Na) in the cooking liquor [146].

The upturn of dissolving wood pulps in the market during the last 10 years may be attributed to a consistent growth of regenerated cellulose fibre production, particularly in China, where 61% of the current global rayon production capacities are located. The annual dissolving wood pulp production in 2011 was 4.2 million t, of which 2.9 million t accounted for commodity applications, e.g., rayon, while the residual 1.3 million t were converted to specialties, e.g., to cellulose acetate. Market studies clearly indicate that this trend of increasing demand of regenerated cellulose fibres and thus dissolving pulps will prevail during the next decades [163]. In the case of sulphite pulping, the annual production of bleached sulphite eucalypt pulp is around one million tonnes per year, contributing to the economic profits of South Africa, Portugal, and Spain [167].

The major components of the spent liquors from sulphite, SEW, and SPORL are lignosulphonates and sugars, which are recognised valuable byproducts for the production of added-value products [164,168,169]. The chemical composition depends on the wood species used for the pulping and this information is essential regarding eventual liquor utilization for different purposes [167,170,171]. Typical spent sulphite liquor from *Eucalyptus globulus* contains lignosulphonates, from 60 to 80 g/L, and sugars, from 35 to 45 g/L, from hydrolysed hemicelluloses, mainly xylose. Hence, this lignocellulosic material is a prospective substrate for bioprocessing once it has a high concentration of monomeric sugars and some proportion of oligomeric saccharides [167]. However, the presence of high amounts of acetic acid (8–9 g·L^−1^), furfural, polyphenols, and low molecular weight lignosulphonates inhibits the microbial metabolism, which is the main drawback for spent sulphite liquor bioprocessing [31,37]. These products of hemicellulose and lignin degradation negatively affect fermentation efficiency due to their toxicity towards fermentative microorganisms, inhibiting both growth and alcoholic metabolisms [37]. Spent sulphite liquors can be considered as promising raw materials for the production of bioethanol since 90 billion litres of spent liquors are produced annually [171]. However, the removal of inhibitors is a difficult task in this kind of samples [31,172,173].

Table 2 shows the results of detoxification in this kind of materials [108,146,162,172,174,175,176,177,178,179,180]. The most problematic task in this case is the separation of lignosulphonates and sugars because lignosulphonates act as a glue in the mixture and the majority of the processes give the same quantity of separation for both lignosulphonates and sugars [174,179]. In the case of sulphite liquor, better results are obtained when using ion exchange resin [175] (with removals of lignosulphonates and acetic acid close to 100% and losses of sugar of 28%) and the best results have been obtained when a combination of processes of overliming, neutralisation with CO_2_, and resin are used [179]. In the case of SPORL liquid, overliming has been used to separate both sugars and lignosulphonates, obtaining the maximum separation when 10 g/L Ca(OH)_2_ at 30 °C and pH equal to 10 during 90 min is used [162]. Membrane operations have also been used to detoxify the samples, giving the best results when combining different membranes in series [178]. Regarding the SEW process, a combination of separation processes in series was necessary to detoxify the samples [146].

In addition to lignosulphonates, other inhibitors can be separated from the spent liquors, such as phenolics, with a high antioxidant activity [180], weak acids, and furans. In this case, the use of exchange resins or the use of liquid–liquid extraction can be very promising techniques [108,180]. A high removal of furans and phenolics and small loss of sugars were found in the results of extraction with chloroform according to the results of Llano et al. [108]. Based on the experiments carried out by Alexandri et al. [180], the use of ethyl acetate at pH equal to 3.4 gives separation close to 100% for phenolics and results in very attractive materials with high antioxidant activity.

Other inhibitors can be metals and SO_2_. In the case of SEW samples, the measured concentration of SO_2_ in the liquors was low because significant SO_2_ losses to the atmosphere occur during the handling of the liquors and the solids. It was shown that all free SO_2_ could be recovered by distillation. The SO_2_ concentration was 101/87 ppm in the liquor, decreasing to 20/25 ppm in the liquor treated with lime, and finally decreasing to 10 ppm/non detect levels. In the liquor treated with the last operation, catalytic oxidation was observed [146]. In addition, other acids such as glucuronic, galacturonic, and 4-*O*-Me-glucuronic acid should be taken into account. In these cases, all of the detoxification treatments carried out by Sklavounos et al. [146] gave removals up to 30% in the case of softwood biomass and an increase of the concentration of these acids in spruce liquor was shown.

## 5. Conclusions

Many inhibitors in lignocellulosic materials are obtained from the pretreatment and they can be grouped into weak acids, furans, phenolics, and others such as SO_2_, lignosulphonates, metals, and extractives. The influence of these compounds in fermentation depends not only on the kind of inhibitor but also on the synergistic or antagonistic effects. However, the removal or separation of these compounds depends more on the kind of inhibitor and the lignocellulosic material. In this work, different physico-chemical processes have been analysed in order to separate the main inhibitors. In addition, most of these components can be used as byproducts for future biorefineries; therefore, a good separation is of great importance.

Overliming can be used to remove levulinic or formic acids, furans, and phenolics; however, this method does not remove acetic acids. In all cases, Ca(OH)_2_ is recommended; however, an optimisation of the pH, between 10 and 11, and reaction time should be done, depending on the inhibitor and the raw material, due to the compromise between the removal of inhibitors and the losses of sugars. The results of detoxification of furans and phenolics depend strongly on the lignocellulosic material when overliming is used.

Adsorption is an attractive and simple solution to detoxify lignocellulosic materials. Activated charcoal is the most used adsorbent; furthermore, other waste materials such as wood charcoal can be used with very good results. Adsorption can be used to remove furans and phenolics and the kind of lignocellulosic material does not have a great influence on the process. Cationic and anionic resins are focused on detoxifying levulinic acid, furans, and phenolics, and maintaining the amount of sugar substrate in the samples. However, an optimisation of this method in the case of separating acetic or formic acids is necessary.

Regarding liquid–liquid extraction, both ethyl acetate and trialkylamine are recommended for furans and phenolics and trialkylamine and trichloroethylene in the case of weak acids, including acetic acid. In addition, cloud point extraction can be used for phenolics.

Membrane operations can be used as a detoxification step. In this case, filtration methods such as ultrafiltration, nanofiltration, and reverse osmosis have been used in the literature, especially for lignin derivatives, giving different results depending on the raw material, with better results in the case of olive residue hydrolysates and synthetic hydrolysates. However, a high fouling tendency of the complex structure of lignocellulosic materials might lead to an inefficient process operation and increased costs. In this case, some pretreatments or other membrane processes such as electrodialysis or resin-wafer electrodeionisation are recommended.

Other processes such as stream stripping, reducing agents for furans, aqueous two-phase extraction, supercritical extraction, and polyelectrolytic flocculants can be used when acids and/or furans are removed and advanced oxidation processes in the case of more recalcitrant inhibitors such as lignin derivatives or extractives. When the objective is to separate different inhibitors, having several possibilities of valorisation, a combination of methods is recommended.

Finally, due to the increase in the market of high-purity cellulose products in pulp and paper mills, acid sulphite process and other novelty processes such as SPORL and SEW are increasing in research. In addition, the spent liquors obtained in these processes contain not only sugars but also lignosulphonates with several applications. In these cases, it is more difficult to separate the sugar substrate from the rest of inhibitors, mainly because the lignosulphonates act as a glue in the mixture. Depending on the pulping process, overliming, resins, membranes, or a combination of processes are the best processes. On the other hand, from a valorisation point of view, the use of liquid–liquid extraction with ethyl acetate at pH equal to 3.4 is recommended in this paper to separate phenolics with high antioxidant activity in these kinds of materials.

As a future work, techno-economic and environmental analysis of the different detoxification methods should be carried out in order to point out the feasibility of all of these alternatives in future biorefineries.

## Figures and Tables

**Table 1:** Main physico-chemical separation methods for lignocellulosic materials.

Method	Characteristics	Inhibitors	Advantages	Disadvantages
Vacuum evaporation	- Reduce volatile compounds - No previous overliming or neutralisation is recommended - Optimisation for pentoses in relation to hexoses, depending on the lignocellulosic material is needed	Acids and furans	- Lower losses of sugars	- Not good for phenolics
Liming and overliming	- Precipitate toxic compounds with alkali treatment - The use of Ca(OH)_2_ is recommended - Optimisation of time and pH to compromise inhibitors removal and losses of sugars is needed	Levulinic acid, furans	- Some phenolics can also be removed - Cheapest option - No high temperature is necessary	- Not good for acetic acid, depending on the material - Sometimes, high losses of sugars
Adsorption	- Separation of substances with an adsorbent - Activated charcoal is the most common sorbent; however, to reduce the losses of sugars, other sorbents can be used - Optimisation of the initial pH is necessary	Levulinic acid, furans and phenolics	- No high temperature - Ease of regeneration and valorisation options	- High losses of sugars in most of cases
Ion exchange resins	- Separation of substances by ion exchange - Both anionic and cationic are recommended to remove all of the inhibitors - Optimisation of the initial pH in the case of acids and furans	Acids, furans, phenolics, heavy metals	- Removal of all of the inhibitors - Ease of regeneration and valorisation options	- High losses of sugars in some cases - Costs of the resins
Liquid–liquid extraction	- Ethyl acetate and trialkylamine for furans and phenolics - Trialkylamine and trichloroethylene for acids - Cloud point extraction in the case of phenolics	Acids, furans, and phenolics	- Ease of regeneration and valorisation options	- Organic solvent management
Filtration by membranes operations	- Microfiltration, nanofiltration, and ultrafiltration - Previous pre-treatment to reduce the fouling	Lignin compounds	- Easy separation and valorisation options	- Fouling problems - Optimisation of the sugar losses is needed

**Table 2:** Separation of lignocellulosic inhibitors in SO_2_-based processes.

Raw Material	Treatment	Inhibitor	Removal (%)	Initial Concentration	Ref.
Hydrolysates from SO_2_-pretreated spruce wood	Activated charcoal	Furans	94	1 g/L	[172]
		Acetic acid	28	1.72 g/L	
		Formic acid	39	0.18 g/L	
		Phenolics	88	1.3 g/L	
	Overliming	Furans	45	1 g/L	
		Aliphatic acids	–	–	
		Phenolics	14	1.3 g/L	
	NH_4_OH	Furans	15	1 g/L	
		Aliphatic acids	–	–	
		Phenolics	8	1.3 g/L	
	NaOH	Furans	8	1 g/L	
		Aliphatic acids	6	1.9 g/L	
		Phenolics	1	1.3 g/L	
	Anion exchanger at pH 10	Furans	26	1 g/L	
		Aliphatic acids	23	1.9 g/L	
		Phenolics	79	1.3 g/L	
	Anion exchanger at pH 5.5	Furans	9	1 g/L	
		Aliphatic acids	28	1.9 g/L	
		Phenolics	53	1.3 g/L	
	Cation exchanger at pH 10	Furans	15	1 g/L	
		Aliphatic acids	10	1.9 g/L	
		Phenolics	22	1.3 g/L	
	Cation exchanger at pH 5.5	Furans	6	1 g/L	
		Aliphatic acids	9	1.9 g/L	
		Phenolics	8	1.3 g/L	
Spent sulphite liquor	Nanofiltration	Lignosulphonates	99	84 g/L	[174]
		Glucose	85	9.31 g/L	
		Xylose	78	30.9 g/L	
	Ultrafiltration	Lignosulphonates	57	84 g/L	
		Sugars	76	49 g/L	
	Reverse Osmosis	Lignosulphonates	68	84 g/L	
		Glucose	96	9.31 g/L	
		Xylose	93	30.9 g/L	
Spent sulphite liquor	Cation and anion exchange	Ca^2+^	99	0.05%	[175]
		Mg^2+^	100	0.55%	
		Lignosulphonates	99	12%	
		Acetic acid	100	1%	
		Sugars	28	5%	
SPORL liquid	10 g/L lime 30 °C pH = 10 90 min	Lignosulphonates	11	50 g/L	[162]
		Glucose	100	12 g/L	
		Xylose	100	148 g/L	
	20 g/L lime 30 °C pH = 12 90 min	Lignosulphonates	26	50 g/L	
		Glucose	100	12 g/L	
		Xylose	60	148 g/L	
	90 g/L lime 30 °C pH = 12.5 90 min	Lignosulphonates	38	50 g/L	
		Glucose	59	12 g/L	
		Xylose	58	148 g/L	
	20 g/L lime 75 °C pH = 12 90 min	Lignosulphonates	36	50 g/L	
		Glucose	21	12 g/L	
		Xylose	5	148 g/L	
Spent sulphite liquor	Ultrafiltration 100 kDa	Lignosulphonates	80	38%	[176]
Spent sulphite liquor	Ultrafiltration	Lignosulphonates	67	56%	[177]
		Sugars	95	32%	
		Acetic acid	36	6.6 g/L	
Spent sulphite liquor	Ultrafiltration 15 kDa/5 kDa in series	Sugars	89	23.31 g/L	[178]
		Lignosulphonates	65	41.1 g/L	
	Ultrafiltration 15 kDa/5 kDa/1 kDa in series	Sugars	82	23.31 g/L	
		Lignosulphonates	72	41.1 g/L	
		Phenolics	76	1.33 g/L	
Spent sulphite liquor	Anionic resin, 24 h, 30 °C, 150 rpm	Acetic acid	10	11.2 g/L	[179]
		Sugars	12	35.9 g/L	
		Lignosulphonates	41	119.5 g/L	
	Overliming CaO pH = 11.5, 70 °C, 15 min	Acetic acid	−19	11.2 g/L	
		Sugars	4	35.9 g/L	
		Lignosulphonates	43	119.5 g/L	
		SO_2_	87	5.5 g/L	
	Activated carbon, 24 h, 30 °C, 150 rpm	Acetic acid	50	11.2 g/L	
		Sugars	6	35.9 g/L	
		Lignosulphonates	20	119.5 g/L	
		SO_2_	−13	5.5 g/L	
	Combined: CaO + resin PA408	Acetic acid	40	11.2 g/L	
		Sugars	91	35.9 g/L	
		Lignosulphonates	90	119.5 g/L	
	Combined: CaO + neutralisation with CO_2_ + resin	Acetic acid	−19	11.2 g/L	
		Sugars	4	35.9 g/L	
		Lignosulphonates	81	119.5 g/L	
SEW Softwood	Evaporation	Sugars	26	18.9%	[146]
	Steam Stripping		+1 *		
	Lime		+2 *		
	Catalytic Oxidation		+2 *		
	Evaporation	Lignin	75	16.7%	
	Steam Stripping		+1 *		
	Lime		+8 *		
	Catalytic Oxidation		+1 *		
	Evaporation	Furfural	99	0.1%	
	Evaporation	HMF	0	0.1 g/L	
	Evaporation	Acetic acid	−100	0.3%	
	Steam Stripping		+100 *		
SEW Spruce	Evaporation	Sugars	6	17.1%	
	Steam Stripping		+2 *		
	Lime		+2 *		
	Catalytic Oxidation		+0 *		
	Evaporation	Lignin	77	17.8%	
	Steam Stripping		+2 *		
	Lime		+0 *		
	Catalytic Oxidation		+4 *		
	Evaporation	Furfural	99	0.2%	
	Evaporation	HMF	0 *	0.1%	
	Evaporation	Acetic acid	60	1.0%	
	Steam Stripping		+40 *		
Spent sulphite liquor	Liquid–liquid extraction with diethyl ether	Sugars	99	193 g/L	[108]
		Phenolics	49	12.40 g/L	
		Acetic acid	58	6.93 g/L	
		Levulinic acid	64	0.11 g/L	
		Formic acid	94	0.23 g/L	
		Furfural	88	0.20 g/L	
		HMF	81	0.13 g/L	
	Liquid–liquid extraction with chloroform	Sugars	99	193 g/L	
		Phenolics	56	12.40 g/L	
		Acetic acid	75	6.93 g/L	
		Levulinic acid	75	0.11 g/L	
		Formic acid	97	0.23 g/L	
		Furfural	97	0.20 g/L	
		HMF	92	0.13 g/L	
Spent sulphite liquor	Ethyl acetate pH 3.4	Phenolics	89–100	12.4 g/L	[180]
	Ethyl acetate pH 2	Phenolics	67–73	12.4 g/L	

* all of the processes are in series; therefore, this is the removal in relation to the previous one, according to the initial concentration.

## References

[1] Ragauskas A.J., Williams C.K., Davison B.H., Britovsek G., Cairney J., Eckert C.A., Frederick W.J., Hallett J.P., Leak D.J., Liotta C.L. (2006). The path forward for biofuels and biomaterials. Science.

[2] Wierckx N., Koopman F., Ruijssenaars H.J., de Winde J.H. (2011). Microbial degradation of furanic compounds: Biochemistry, genetics, and impact. Appl. Microbiol. Biotechnol..

[3] Silva J.P.A., Carneiro L.M., Roberto I.C. (2013). Treatment of rice straw hemicellulosic hydrolysates with advanced oxidative processes: A new and promising detoxification method to improve the bioconversion process. Biotechnol. Biofuels.

[4] Saini J.K., Saini R., Tewari L. (2015). Lignocellulosic agriculture wastes as biomass feedstocks for second-generation bioethanol production: Concepts and recent developments. Biotechnology.

[5] Rowell R.M., Waldron K. (2014). The use of biomass to produce bio-based composites and building materials. Advances in Biorefineries. Biomass and Waste Supply Chain Exploitation.

[6] Fava F., Totaro G., Diels L., Reis M., Duarte J., Carioca O.B., Poggi-Varaldo H.M., Sommer Ferreira B. (2015). Biowaste biorefinery in Europe: Opportunities and research & development needs. New Biotechnol..

[7] Saha B.C. (2003). Hemicellulose bioconversion. J. Ind. Microbiol. Biotechnol..

[8] Liu Z.L., Slininger P.J., Gorsich S.W. (2005). Enhanced biotransformation of furfural and hydroxymethylfurfural by newly developed ethanologenic yeast strains. Appl. Biochem. Biotechnol..

[9] Okuda N., Soneura M., Ninomiya K., Katakura Y., Shioya S. (2008). Biological detoxification of waste house wood hydrolysate using *Urebacillus* thermosphaericus for bioethanol production. J. Biosci. Bioeng..

[10] Lee J.M., Venditti R.A., Jameel H., Kenealy W.R. (2011). Detoxification of woody hydrolyzates with activated carbon for bioconversion to ethanol by the thermophilic anaerobic bacterium *Thermoanaerobacterium* saccharolyticum. Biomass Bioenergy.

[11] Parawira W., Tekere M. (2011). Biotechnological strategies to overcome inhibitors in lignocellulose hydrolysates for ethanol production: Review. Crit. Rev. Biotechnol..

[12] Sarkar N., Ghosh S.K., Bannerjee S., Aikat K. (2012). Bioethanol production from agricultural wastes: An overview. Renew. Energy.

[13] Sixta H. (2006). Handbook of Pulp.

[14] Huang H.-J., Ramaswamy S.H., Tschirner U.W., Ramarao B.V. (2008). A review of separation technologies in current and future biorefineries. Sep. Purif. Technol..

[15] Boussarsar H., Rogé B., Mathlouthi M. (2009). Optimization of sugarcane bagasse conversion by hydrothermal treatment for the recovery of xylose. Bioresour. Technol..

[16] Llano T., Rueda C., Quijorna N., Blanco A., Coz A. (2012). Study of the delignification of hardwood chips in a pulping process for sugar production. J. Biotechnol..

[17] Lee H.-J., Lim W.-S., Lee J.-W. (2013). Improvement of ethanol fermentation from lignocellulosic hydrolysates by the removal of inhibitors. J. Ind. Eng. Chem..

[18] Zhou F., Wanga C., Wei J. (2013). Simultaneous acetic acid separation and monosaccharide concentration by reverse osmosis. Bioresour. Technol..

[19] Bref Document—IPPC (2015). Reference Document on Best Available Techniques in the Pulp and Paper Industry.

[20] Toledano A., Serrano L., Garcia A., Mondragon I., Labidi J. (2010). Comparative study of lignin fractionation by ultrafiltration and selective precipitation. Chem. Eng. J..

[21] Chandel A.K., Singh O.V., Rao L.V., Singh O.V., Harvey S.P. (2010). Biotechnological applications of hemicellulosic derived sugars: State-of-the-art. Sustainable Biotechnology: Renewable Resources and New Perspectives.

[22] Chandel A.K., da Silva S.S., Singh O.V. (2013). Detoxification of lignocellulose hydrolysates: Biochemical and metabolic engineering toward white biotechnology. Bioenergy Res..

[23] Holladay J.E., Bozell J.J., White J.F., Johnson D. (2007). Top Value Added Chemicals from Biomass: Volume II—Results of Screening for Potential Candidates from Biorefinery Lignin.

[24] Zhang X., Tu M., Paice M.G. (2011). Routes to potential bioproducts from lignocellulosic biomass lignin and hemicelluloses. Bioenergy Res..

[25] Bizzari S.N., Janshekar H., Yokose K. (2010). Lignosulfonates.

[26] IEA IEA Bioenergy Annual Report 2009. http://www.ieabioenergy.com/wp-content/uploads/2013/10/IEA-Bioenergy-2009-Annual-Report.pdf.

[27] Moshkelani M., Marinova M., Perrier M., Paris J. (2013). The forest biorefinery and its implementation in the pulp and paper industry: Energy overview. Appl. Therm. Eng..

[28] Agbor V., Carere C., Cicek N., Sparling R., Levin D., Waldron K. (2014). Biomass pretreatment for consolidated bioprocessing (CBP). Advances in Biorefineries. Biomass and Waste Supply Chain Exploitation.

[29] Harmsen P., Huijgen W., Bermudez L., Bakker R. (2010). Literature review of physical and chemical pretreatment processes for lignocellulosic biomass. Biosinergy.

[30] Lenihan P., Orozco A., O’Neill E., Ahmad M.N.M., Rooney D.W., Walker G.M. (2010). Dilute acid hydrolysis of lignocellulosic biomass. Chem. Eng. J..

[31] Xavier A.M.R.B., Correia M.F., Pereira S.R., Evtuguin D.V. (2010). Second-generation bioethanol from eucalypt sulphite spent liquor. Bioresour. Technol..

[32] Liu X., Lu M., Ai N., Yu F., Ji J. (2012). Kinetic model analysis of dilute sulfuric acid-catalyzed hemicellulose hydrolysis in sweet sorghum bagasse for xylose production. Ind. Crops Prod..

[33] Martín M., Grossmann I.E. (2013). Review: On the systematic synthesis of sustainable biorefineries. Ind. Eng. Chem. Res..

[34] Liu Z.L. (2006). Genomic adaptation of ethanologenic yeast to biomass conversion inhibitors. Appl. Microbiol. Biotechnol..

[35] Palmqvist E., Hahn-Hägerdal B. (2000). Fermentation of lignocellulosic hydrolysates I: Inhibition and detoxification. Bioresour. Technol..

[36] Palmqvist E., Hahn-Hägerdal B. (2000). Fermentation of lignocellulosic hydrolysates II: Inhibitors and mechanism of inhibition review. Bioresour. Technol..

[37] Mussatto S.I., Roberto I.C. (2004). Alternatives for detoxification of diluted-acid lignocellulosic hydrolyzates for use in fermentative processes: A review. Bioresour. Technol..

[38] Chandel A.K., Silva S.S., Singh O.V., Bernardes M.A.S. (2011). Detoxification of lignocellulosic hydrolysates for improved bioconversion of bioethanol. Biofuel Production—Recent Developments and Prospects.

[39] Holm-Nielsen J.B., Ehimen E.A., Waldron K. (2014). Biorefinery plant design, engineering and process optimization. Advances in Biorefineries. Biomass and Waste Supply Chain Exploitation.

[40] Pampulha M.E., Loureiro-Dias M.C. (1990). Activity of glucolytic enzymes of *Saccharomyces cerevisiae* in the presence of acetic acid. Appl. Microbiol. Biotechnol..

[41] Zaldivar J., Ingram L.O. (1999). Effect of organic acids on the growth and fermentation of ethanologenic *Escherichia coli* LY01. Biotechnol. Bioeng..

[42] Klinke H.B., Thomsen A.B., Ahring B.K. (2004). Inhibition of ethanol producing yeast and bacteria by degradation products produced during pretreatment of biomass. Appl. Microb. Biotechnol..

[43] Gurram R.N., Datta S., Lin Y.J., Snyder S.W., Menkhaus T.J. (2011). Removal of enzymatic and fermentation inhibitory compounds from biomass slurries for enhanced biorefinery process efficiencies. Bioresour. Technol..

[44] Palmqvist E., Grage H., Meinander N.Q., Hahn-Hägerdal B. (1999). Main and interaction effects of acetic acid, furfural, and *p*-hydroxybenzoic acid on growth and ethanol productivity of yeasts. Biotechnol. Bioeng..

[45] Zaldivar J., Martínez A., Ingram L.O. (1999). Effect of selected aldehydes on the growth and fermentation of ethanologenic *Escherichia coli*. Biotechnol. Bioeng..

[46] Zaldivar J., Marínez A., Ingram L.O. (2000). Effect of alcohol compounds found in hemicellulose hydrolysate on the growth and fermentation of ethanologenic *Escherichia coli*. Biotechnol. Bioeng..

[47] Helle S., Cameron D., Lam J., White B., Duff S. (2003). Effect of inhibitory compounds found in biomass hydrolysates on growth and xylose fermentation by a genetically engineered strain of *S. cerevisiae*. Enzyme Microb. Technol..

[48] Liu Z.L., Slininger P.J., Dien B.S., Berhow M.A., Kurtzman C.P., Gorsich S.W. (2004). Adaptive response of yeasts to furfural and 5-hydroxymethylfurfural and new chemical evidence for HMF conversion to 2,5-bis-hydroxymethylfuran. J. Ind. Microbiol. Biotechnol..

[49] Pienkos P.T., Zhang E.M. (2009). Role of pretreatment and conditioning processes on toxicity of lignocellulosic biomass hydrolysates. Cellulose.

[50] Carvalho G.B.M., Mussatto S.I., Cândido E.J., Almeida e Silva J.B. (2006). Comparison of different procedures for the detoxification of eucalyptus hemicellulosic hydrolysate for use in fermentative processes. J. Chem. Technol. Biotechnol..

[51] Wang B., Feng H., Blaschek H.P., Ezeji T., Scheffran J. (2010). Detoxification of lignocellulosic hydrolysates. Biofuels from Agricultural Wastes and Byproducts.

[52] Qi B., Luo J., Chen X., Hang X., Wan Y. (2011). Separation of furfural from monosaccharides by nanofiltration. Bioresour. Technol..

[53] Canilha L., Chandel A.K., dos Santos Milessi T.S., Fernandes Antunes F.A., da Costa Freitas W.L., Almeida Felipe M.G., da Silva S.S. (2012). Review article: Bioconversion of sugarcane biomass into ethanol: An overview about composition, pretreatmentmethods, detoxification of hydrolysates, enzymatic saccharification, and ethanol fermentation. J. Biomed. Biotechnol..

[54] Larsson S., Reimann A., Nilvebrant N.O., Jonsson L.J. (1999). Comparison of different methods for the detoxification of lignocellulose hydrolyzates of spruce. Appl. Biochem. Biotechnol..

[55] Mussatto S.I., Santos J.C., Roberto I.C. (2004). Effect of pH and activated charcoal adsorption on hemicellulosic hydrolysate detoxification for xylitol production. J. Chem. Technol. Biotechnol..

[56] Horváth I., Sjöde A., Alriksson B., Jönsson L., Nilvebrant N.-O. (2005). Critical conditions for improved fermentability during overliming of acid hydrolysates from spruce. Appl. Biochem. Biotechnol..

[57] Chandel A.K., Kapoor R.K., Singh A., Kuhad R.C. (2007). Detoxification of sugarcane bagasse hydrolysate improves ethanol production by *Candida shehatae* NCIM 3501. Bioresour. Technol..

[58] Telli-Okur M., Eken-Saraçoglu N. (2008). Fermentation of sunflower seed hull hydrolysate to ethanol by *Pichia stipitis*. Bioresour. Technol..

[59] Liu Z.L., Blaschek H.P., Vertès A.A., Qureshi N., Blaschek H.P., Yukawa H. (2010). Biomass conversion inhibitors and in situ detoxification. Biomass to Biofuels: Strategies for Global Industries.

[60] Carter B., Squillace P.H., Gilcrease P.C., Menkhaus T.J. (2011). Detoxification of a lignocellulosic biomass slurry by soluble polyelectrolyte adsorption for improved fermentation efficiency. Biotechnol. Bioeng..

[61] Tengborg C., Galbe M., Zacchi G. (2001). Reduced inhibition of enzymatic hydrolysis of steam-pretreated softwood. Enzyme Microb. Technol..

[62] Villarreal M.L.M., Prata A.M.R., Felipe M.G.A., Almeida E., Silva J.B. (2006). Detoxification procedures of eucalyptus hemicellulose hydrolysate for xylitol production by *Candida guilliermondii*. Enzyme Microb. Technol..

[63] Zhu J., Yong Q., Xu Y., Yu S.H. (2011). Detoxification of corn stover prehydrolyzate by trialkylamine extraction to improve the ethanol production with *Pichia stipitis* CBS 5776. Bioresour. Technol..

[64] Sierra-Alvarez R., Lettinga G. (1991). The methanogenic toxicity of wastewater lignins and lignin related compounds. J. Chem. Technol. Biotechnol..

[65] Larsson S., Palmqvist E., Hahn-Hägerdal B., Tengborg C., Stenberg K., Zacchi G., Nilvebrant N. (1999). The generation of inhibitors during dilute acid hydrolysis of softwood. Enzyme Microb. Technol..

[66] Lewkowski J. (2001). Synthesis, chemistry and applications of 5-hydroxymethylfurfural and its derivatives. ARKIVOC.

[67] Liu Z.L., Moon J., Andersh B.J., Slininger P.J., Weber S. (2008). Multiple gene mediated aldehyde reduction is a mechanism of in situ detoxification of furfural and HMF by ethanologenic yeast *Saccharomyces cerevisiae*. Appl. Microbiol. Biotechnol..

[68] Heer D., Sauer U. (2008). Identification of furfural as a key toxin in lignocellulosic hydrolysates and evolution of a tolerant yeast strain. Microb. Biotechnol..

[69] Almeida J.R.M., Bertilsson M., Gorwa-Grauslund M.F., Gorsich S., Liden G. (2009). Metabolic effects of furaldehydes and impacts on biotechnological processes. Appl. Microbiol. Biotechnol..

[70] Thomsen M.H., Thygesen A., Thomsen A.B. (2009). Identification and characterization of fermentation inhibitors formed during hydrothermal treatment and following SSF of wheat straw. Appl. Microbiol. Biotechnol..

[71] Olsson L., Hahn-Hägerbal B. (1996). Fermentation of lignocellulosic hydrolysates for ethanol production. Enzyme Microb. Technol..

[72] Taherzadeh M.J., Gustafsson L., Niklasson C., Lindén G. (2000). Physiological effects of 5-hydroxymethylfurfural on *Saccharomyces cerevisiae*. Appl. Microbiol. Biotechnol..

[73] Ezeji T., Qureshi N., Blaschek H. (2007). Butanol production from agricultural residues: Impact of degradation products on *Clostridium beijerinckii* growth and butanol fermentation. Biotechnol. Bioeng..

[74] Banerjee N., Bhatnagar R., Viswanathan L. (1981). Inhibition of glycolysis by furfural in *Saccharomyces cerevisiae*. Eur. J. Appl. Microbiol. Biotechnol..

[75] Jing X., Zhang X., Bao J. (2009). Inhibition performance of lignocellulose degradation products on industrial cellulase enzymes during cellulose hydrolysis. Appl. Biochem. Biotechnol..

[76] Taherzadeh M.J., Gustafsson L., Niklasson C., Lidén G. (1999). Conversion of furfural in aerobic and anaerobic batch fermentation of glucose by *Saccharomyces cerevisiae*. J. Biosci. Bioeng..

[77] Meinita M.D.N., Hong Y.-K., Jeong G.-T. (2012). Detoxification of acidic catalyzed hydrolysate of *Kappaphycus alvarezii* (cottonii). Bioprocess Biosyst. Eng..

[78] Lin A.S., Qian K., Usami Y., Lin L., Itokawa H., Hsu C., Morris-Natschke S.L., Lee K.H. (2008). 5-Hydroxymethyl-2-furfural, a clinical trials agent for sickle cell anemia, and its mono/di-glucosides from classically processed steamed rehmanniae radix. J. Nat. Med..

[79] Ding X., Wang M.Y., Yao Y.X., Li G.Y., Cai B.C. (2010). Protective effect of 5-hydroxymethylfurfural derived from processed *Fructus Corni* on human hepatocyte LO2 injured by hydrogen peroxide and its mechanism. J. Ethnopharmacol..

[80] Michail K., Matzi V., Maier A., Herwig R., Greilberger J., Juan H., Kunert O., Wintersteiger R. (2007). Hydroxymethylfurfural: An enemy or a friendly xenobiotic? A bioanalytical approach. Anal. Bioanal. Chem..

[81] Almeida J.R.M., Modig T., Petersson A., Hahn-Hägerdal B., Liden G., Gorwa-Grauslund M.F. (2007). Increased tolerance and conversion of inhibitors in lignocellulosic hydrolysates by *Saccharomyces cerevisiae*. J. Chem. Technol. Biotechnol..

[82] Taherzadeh M.J., Niklasson C., Lidén G. (1997). Acetic-acid—Friend or foe in anaerobic batch conversion of glucose to ethanol by *Saccharomyces cerevisiae*?. Chem. Eng. Sci..

[83] Heipieper J.J., Weber F.J., Sikkema J., Keweloh H., de Bont J.A.M. (1994). Mechanism of resistance of whole cells to toxic organic solvents. Trends Biotechnol..

[84] Odeh R.M., Cornish L.A. (1995). Natural antioxidants for the prevention of atherosclerosis. Pharmacotherapy.

[85] German J.B., Walzem R.L. (2000). The health benefits of wine. Annu. Rev. Nutr..

[86] Erlund I. (2004). Review of the flavonoids quercetin, hesperetin, and naringenin. Dietary sources, bioactivities, bioavailability, and epidemiology. Nutr. Res..

[87] Seifried H.E., Anderson D.E., Fisher E.I., Milner J.A. (2007). A review of the interaction among dietary antioxidants and reactive oxygen species. J. Nutr. Biochem..

[88] Bonfili L., Cecarini V., Amici M., Cuccioloni M., Angeletti M., Keller J.N., Eleuteri A.M. (2008). Natural polyphenols as proteasome modulators and their role as anti-cancer compounds. FEBS J..

[89] Middleton E.J., Kandaswami C., Theoharides T.C. (2000). The effects of plant flavonoids on mammalian cells: Implications for inflammation, heart disease, and cancer. Pharmacol. Rev..

[90] Havsteen B.H. (2002). The biochemistry and medical significance of the flavonoids. Pharmacol. Ther..

[91] Halliwell B. (2009). The wanderings of a free radical. Free Radic. Biol. Med..

[92] Soto M.L., Moure A., Dominguez H., Parajó J.C. (2011). Recovery, concentration and purification of phenolic compounds by adsorption: A review. J. Food Eng..

[93] Martin C., Jönsson L. (2003). Comparison of the resistance of industrial and laboratory strains of *Saccharomyces* and *Zygosaccharomyces* to lignocellulose-derived fermentation inhibitors. Enzyme Microb. Technol..

[94] Nigam J.N. (2001). Ethanol production from wheat straw hemicelluloses hydrolysate by *Scheffersomyces stipitis*. J. Biotechnol..

[95] Kumar P., Barrett D.M., Delwiche M.J., Stroeve P. (2009). Methods for pretreatment of lignocellulosic biomass for efficient hydrolysis and biofuel production. Ind. Eng. Chem. Res..

[96] Martinez A., Rodriguez M.E., Wells M.L., York S.W., Preston J.F., Ingram L.O. (2001). Detoxification of dilute acid hydrolysates of lignocellulose with lime. Biotechnol. Prog..

[97] Martínez-Patiño J.C., Romero-García J.M., Ruiz E., Oliva J.M., Álvarez C., Romero I., Negro M.J., Castro E. (2015). High solids loading pretreatment of olive tree pruning with dilute phosphoric acid for bioethanol production by *Escherichia coli*. Energy Fuels.

[98] Nilvebrant N.-O., Reimann A., Larsson S., Jönsson L.J. (2001). Detoxification of lignocellulose hydrolysates with ion-exchange resins. Appl. Biochem. Biotechnol..

[99] Alves L.A., Vitolo M., Felipe M.G.A., Almeida e Silva J.B. (2002). Xylose reductase and xylitol dehydrogenase activities of *Candida guilliermondii* as a function of different treatments of sugarcane bagasse hemicellulosic hydrolysate employing experimental design. Appl. Biochem. Biotechnol..

[100] Weil J.R., Dien B., Bothast R., Hendrickson R., Mosier N.S., Ladisch M.R. (2002). Removal of fermentation inhibitors formed during pretreatment of biomass by polymeric adsorbents. Ind. Eng. Chem. Res..

[101] Carvalho W., Canilha L., Mussatto S.I., Dragone G., Morales M.L.V., Solenzal A.I.N. (2004). Detoxification of sugarcane bagasse hemicellulosic hydrolysate with ion-exchange resins for xylitol production by calcium alginate-entrapped cells. J. Chem. Technol. Biotechnol..

[102] Carvalho R.J., Marton J.M., Silva F., Felipe M.G. (2005). Avaliação do sistema combinado de tratamento do hidrolisado hemicelulósico de bagaço de cana-de-açúcar com carvão ativo e resinas de troca iônica para sua utilização como meio de fermentação. Rev. Anal..

[103] Xie Y., Phelps D., Lee C.-H., Sedlak M., Ho N., Wang N.-H.L. (2005). Comparison of two adsorbents for sugar recovery from biomass hydrolyzate. Ind. Eng. Chem. Res..

[104] Ranjan R.S., Thust S., Gounaris C.E., Woo M., Floudas C.A., von Keitz M., Valentas K.J., Wei J., Tsapatsis M. (2009). Adsorption of fermentation inhibitors from lignocellulosic biomass hydrolyzates for improved ethanol yield and valueadded product recovery. Microporous Mesoporous Mater..

[105] Zhuang J., Liu Y., Wu Z., Sun Y., Lin L. (2009). Hydrolysis of wheat straw hemicellulose and detoxification of the hydrolysate for xylitol production. BioResources.

[106] Lopez-Linares J.C., Cara-Corpas C., Ruiz-Ramos E., Moya-Vilar M., Castro-Galiano E., Romero-Pulido I. (2015). Hemicellulose-derived sugars solubilisation of rape straw. Cofermentation of pentoses and hexoses by *Escherichia coli*. Span. J. Agric. Res..

[107] Christopher M., Anusree M., Mathew A.K., Nampoothiri K.M., Sukumaran R.K., Pandey A. (2016). Detoxification of acidic biorefinery waste liquor for production of high value amino acid. Bioresour. Technol..

[108] Llano T., Alexandri M., Koutinas A., Gardeli C.H.R., Papapostolou H., Coz A., Quijorna N., Andres A., Komaitis M. (2015). Liquid-liquid extraction of phenolic compounds from spent sulphite liquor. Waste Biomass Valor.

[109] Converti A., Domínguez J.M., Perego P., Silva S.S., Zilli M. (2000). Wood hydrolysis and hydrolysate detoxification for subsequent xylitol production. Chem. Eng. Technol..

[110] Rodrigues R.C.L.B., Felipe M.G.A., Almeida e Silva J.B., Vitolo M., Gómez P.V. (2001). The influence of pH, temperature and hydrolyzate concentration on the removal of volatile and nonvolatile compounds from sugarcane bagasse hemicellulosic hydrolyzate treated with activated charcoal before or after vacuum evaporation. Braz. J. Chem. Eng..

[111] López M.J., Nichols N.N., Dien B.S., Moreno J., Bothast R.J. (2004). Isolation of microorganisms for biological detoxification of lignocellulosic hydrolysates. Appl. Microbiol. Biotechnol..

[112] Nichols N.N., Dien B.S., Cotta M.A. (2010). Fermentation of bioenergy crops into ethanol using biological abatement for removal of inhibitors. Bioresour. Technol..

[113] Alriksson B., Cavka A., Jönsson L.J. (2011). Improving the fermentability of enzymatic hydrolysates of lignocelluloses through chemical in-situ detoxification with reducing agents. Bioresour. Technol..

[114] Anish R., Rao M., Pandey A. (2009). Bioethanol from lignocellulosic biomass part III hydrolysis and fermentation. Handbook of Plant-Based Biofuels.

[115] Sainio T., Turku I., Heinonen J. (2011). Adsorptive removal of fermentation inhibitors from concentrated acid hydrolyzates of lignocellulosic biomass. Bioresour. Technol..

[116] Schirmer-Michel Â.C., Flôres S.H., Hertz P.F., Matos G.S., Ayub M.A.Z. (2008). Production of ethanol from soybean hull hydrolysate by osmotolerant *Candida guilliermondii* NRRL Y-2075. Bioresour. Technol..

[117] Huang C.H., Zong M.-H., Wu H., Liu Q.-P. (2009). Microbial oil production from rice straw hydrolysate by *Trichosporon fermentans*. Bioresour. Technol..

[118] Mateo S., Roberto I.C., Sánchez S., Moya A.J. (2013). Detoxification of hemicellulosic hydrolyzate from olive tree pruning residue. Ind. Crops Prod..

[119] Carvalheiro F., Duarte L.C., Lopes S., Parajó J.C., Pereira H., Gírio F.M. (2005). Evaluation of the detoxification of brewery’s spent grain hydrolysate for xylitol production by *Debaryomyces hansenii* CCMI 941. Process Biochem..

[120] Millati R., Niklasson C., Taherzadeh M.J. (2002). Effect of pH, time and temperature of overliming on detoxification of dilute-acid hydrolyzates for fermentation by *Saccharomyces cerevisiae*. Proc. Biochem..

[121] Carlos Martín C., Galbe M., Wahlbom C.F., Hahn-Hägerdal B., Jönsson L.J. (2002). Ethanol production from enzymatic hydrolysates of sugarcane bagasse using recombinant xylose-utilising *Saccharomyces cerevisiae*. Enzyme Microb. Technol..

[122] Alriksson B., Sjöde A., Nilvebrant N.O., Jönsson L.J. (2006). Optimal conditions for alkaline detoxification of dilute-acid lignocellulose hydrolysates. Appl. Biochem. Biotechnol..

[123] Andary J., Maalouly J., Ouaini R., Chebib H., Rutledge D.N., Ouaini N. (2012). Application of 2D correlation spectroscopy on olive stones acid hydrolysates: Effect of overliming. Chemom. Intell. Lab..

[124] Purwadi R., Niklasson C., Taherzadeh M.J. (2004). Kinetic study of detoxification of dilute-acid hydrolyzates by Ca(OH)_2_. J. Biotechnol..

[125] Berson R.E., Young J.S., Hanley T.R. (2006). Reintroduced solids increase inhibitor levels in a pretreated corn stover hydrolysate. Appl. Biochem. Biotechnol..

[126] Datta S., Lin Y.J., Snyder S.W., Waldron K. (2014). Current and emerging separations technologies in biorefining. Advances in Biorefineries. Biomass and Waste Supply Chain Exploitation.

[127] Eken-Saraçoglu N., Arslan Y. (2000). Comparison of different pretreatments in ethanol fermentation using corn cob hemicellulosic hydrolysate with *Pichia stipitis* and *Candida shehatae*. Biotechnol. Lett..

[128] Ribeiro M.H.L., Lourenço P.A.S., Monteiro J.P., Ferreira-Dias S. (2001). Kinetics of selective adsorption of impurities from a crude vegetable oil in hexane to activated earths and carbons. Eur. Food Res. Technol..

[129] Miyafuji H., Danner H., Neureiter M., Thomasser C., Bvochora J., Szolar O., Rudolf B. (2003). Detoxification of wood hydrolysates with wood charcoal for increasing the fermentability of hydrolysates. Enzyme Microb. Technol..

[130] De Mancilha I.M., Karim M.N. (2003). Evaluation of ion exchange resins for removal of inhibitory compounds from corn stover hydrolyzate for xylitol fermentation. Biotechnol. Prog..

[131] Griffin G.J., Shu L. (2004). Solvent extraction and purification of sugars from hemicellulose hydrolysates using boronic acid carriers. J. Chem. Technol. Biotechnol..

[132] Zhu J., Zhu Y., Zhang L., Yong Q., Xu Y., Li X., Lian Z., Yu S. (2014). Sodium hydroxide regeneration of trialkylamine extractant containing inhibitors from corn stover prehydrolyzate by liquid-liquid extraction. Sep. Purif. Technol..

[133] Wilson J.J., Deschatelets L., Nishikawa N. (1989). Comparative fermentability of enzymatic and acid hydrolysates of steam-pretreated aspenwood hemicellulose by *Pichia stipitis* CBS 5776. Appl. Microbiol. Biotechnol..

[134] Frazer F.R., McCaskey T.A. (1989). Wood hydrolyzate treatments for improved fermentation of wood sugars to 2,3-butanediol. Biomass.

[135] Dhamole P.B., Wang B., Feng H. (2013). Detoxification of corn stover hydrolysate using surfactant-based aqueous two phase system. J. Chem. Technol. Biotechnol..

[136] Samaddar P., Sen K. (2014). Cloud point extraction: A sustainable method of elemental preconcentration and speciation. J. Ind. Eng. Chem..

[137] Pinelo M., Jonsson G., Meyer A.S. (2009). Membrane technology for purification of enzymatically produced oligosaccharides: Molecular and operational features affecting performance. Sep. Purif. Technol..

[138] Li N.N., Fane A.G., Ho W.S., Matsuura T. (2008). Advanced Membrane Technology and Applications.

[139] Koivula E., Kallioinen M., Preis S., Testova L., Sixta H., Mänttäri M. (2011). Evaluation of various pretreatment methods to manage fouling in ultrafiltration of wood hydrolysates. Sep. Purif. Technol..

[140] Weng Y.-H., Wei H.-J., Tsai T.-Y., Lin T.-H., Wei T.-Y., Guo G.-L., Huang C.H.-P. (2010). Separation of furans and carboxylic acids from sugars in dilute acid rice straw hydrolyzates by nanofiltration. Bioresour. Technol..

[141] Chen J., Zhang Y., Wang Y., Ji X., Zhang L., Mi X., Huang H. (2013). Removal of inhibitors from lignocellulosic hydrolyzates by vacuum membrane distillation. Bioresour. Technol..

[142] Brás T., Guerra V., Torrado I., Lourenço P., Carvalheiro F., Duarte L.C., Neves L.A. (2014). Detoxification of hemicellulosic hydrolysates from extracted olive pomace by diananofiltration. Process Biochem..

[143] Nguyen N., Fargues C., Lewandowsky R., Guiga W., Lameliose M.L. (2012). Assessing nanofiltration and reverse osmosis for the detoxification of fermentable solutions. Proc. Eng..

[144] Kim J.H., Na J.-G., Yang J.-W., Chang Y.K. (2013). Separation of galactose, 5-hydroxymethylfurfural and levulinic acid in acid hydrolysate of agarose by nanofiltration and electrodialysis. Bioresour. Technol..

[145] Wallberg O., Linde M., Jönsson A.-S. (2006). Extraction of lignin and hemicelluloses from kraft black liquor. Desalination.

[146] Sklavounos E., Iakovlev M., van Heiningen A. (2013). Study on conditioning of SO_2_-Ethanol-Water spent liquor from spruce chips/softwood biomass for ABE fermentation. Ind. Eng. Chem. Res..

[147] Parajó J.C., Dominguez H., Dominguez J.M. (1997). Xylitol production from Eucalyptus wood hydrolysates extracted with organic solvents. Process Biochem..

[148] Nilsson A., Gorwa-Grauslund M.F., Hahn-Hägerdal B., Lidén G. (2005). Cofactor dependence in furan reduction by *Saccharomyces cerevisiae* in fermentation of acid-hydrolyzed lingocellulose. Appl. Environ. Microbiol..

[149] Lee J.-W., Trinh L.T.P., Lee H.-J. (2014). Removal of inhibitors from a hydrolysate of lignocellulosic biomass using electrodialysis. Sep. Purif. Technol..

[150] Sreenath H., Jeffries T.W. (2000). Production of ethanol from wood hydrolyzate by yeasts. Bioresour. Technol..

[151] Lin Y.J., Henry M.P., Snyder S.W. (2004). Electronically and Ionically Conductive Porous Material and Method for Manufacture of Resin Wafers Therefrom. U.S. Patent.

[152] Arora M.B., Hestekin J.A., Snyder S.W., Martin E.J., Donnelly M.I., Sanville-Millard C., Lin Y.J. (2007). The separative bioreactor: A continuous separation process for the simultaneous production and direct capture of organic acids. Sep. Sci. Technol..

[153] Lin Y.J., Henry M.P., Snyder S.W., Martin E.S., Arora M.B., de la Garza L. (2005). Devices Using Resin Wafers and Applications Thereof. U.S. Patent.

[154] Datta S., Henry M.P., Ahmad S.F., Snyder S.W., Lin Y.J. Removal of salt impurities from glycerin using electrodeionization technique. Proceedings of the AIChE National Meeting.

[155] Datta S., Lin Y.J., Schell D.J., Millard C.S., Ahmad S.F., Henry M.P., Gillenwater P., Fracaro A.T., Moradia A., Gwarnicki Z.P. (2013). Removal of acidic impurities from corn stover hydrolysate liquor by resin wafer based electrodeionization. Ind. Eng. Chem. Res..

[156] Lin Y.J., Snyder S.W., Trachtenberg M.C., Cowan R.M., Datta S. (2009). Carbon Dioxide Capture Using Resin-Wafer Electrodeionization. U.S. Patent.

[157] Hasmann F.A., Santos V.C., Gurpilhares D.B., Pessoa-Junior A., Roberto I. (2008). Aqueous two-phase extraction using thermoseparating copolymer: A new system for phenolic compounds removal from hemicelullosic hydrolysates. J. Chem. Technol. Biotechnol..

[158] Persson P., Larsson S., Jönsson N.J., Nilvebrant N.-O., Sivik B., Munteanu F., Thörneby L., Gorton L. (2002). Supercritical fluid extraction of a lignocellulosic hydrolysate of spruce for detoxification and to facilitate analysis of inhibitors. Biotechnol. Bioeng..

[159] Ahmed B., Mohamed H., Limem E., Bensalah N. (2009). Degradation and mineralization of organic pollutants contained in actual pulp and paper mill wastewaters by a UV/H_2_O_2_ process. Ind. Eng. Chem..

[160] Carter B., Gilcrease P.C., Menkhaus T.J. (2011). Removal and recovery of furfural, 5-hydroxymethylfurfural, and acetic acid from aqueous solutions using a soluble polyelectrolyte. Biotechnol. Bioeng..

[161] Burke D.R., Anderson J., Gilcrease P.C., Menkhuas T.J. (2011). Enhanced solid-liquid clarification of lignocellulosic slurries using polyelectrolyte flocculating agents. Biomass Bioenergy.

[162] Yu M., Wang G., Liu C., Ruhan A. (2012). Precipitation of lignosulphonates from SPORL liquid by calcium hydroxide treatment. BioResources.

[163] Sixta H., Iakovlev M., Testova L., Roselli A., Hummel M., Borrega M., van Heiningen A., Froschauer C., Schottenberger H. (2013). Novel concepts of dissolving pulp production. Cellulose.

[164] Maican E., Teixeira J.A., Ferdeş M., Coz A. (2015). Energy efficient technologies for lignocellulosic ethanol production. INMATEH Agric. Eng..

[165] Zhu W., Zhu J., Gleisner R., Pan X. (2010). On energy consumption for size-reduction and yields from subsequent enzymatic saccharification of pretreated lodgepole pine. Bioresour. Technol..

[166] Uihlein A., Schebec L. (2009). Environmental impacts of a lignocellulose feedstock biorefinery system: An assessment. Biomass Bioenergy.

[167] Marques A.P., Evtuguin D.V., Magina S., Amado F.M.L., Prates A. (2009). Chemical composition of spent liquors from acidic magnesium-based sulphite pulping of *Eucalyptus globulus*. J. Wood. Chem. Technol..

[168] Hocking M.B. (1997). Vanillin: Synthetic flavoring from spent sulfite liquor. J. Chem. Educ..

[169] Plank J. (2004). Applications of biopolymers and other biotechnological products in building materials. Appl. Microbiol. Biotechnol..

[170] Smook G.A. (2002). Handbook for Pulp & Paper Technologists.

[171] Pereira S.R., Portugal-Nunes D.-N., Evtuguin D.V., Serafim L.S., Xavier A.M.R.B. (2013). Advances in ethanol production from hardwood spent sulphite liquors. Process Biochem..

[172] Guo X., Cavka A., Jönsson L.F., Hong F. (2013). Comparison of methods for detoxification of spruce hydrolysate for bacterial cellulose production. Microb. Cell Fact..

[173] Pereira S.R., Ivanuša Š., Evtuguin D.V., Serafim L.S., Xavier A.M.R.B. (2012). Biological treatment of eucalypt spent sulphite liquors: A way to boost the production of second generation bioethanol. Bioresour. Technol..

[174] Restolho J.A., Prates A., de Pinho M.N., Afonso M.D. (2009). Sugars and lignosulphonates recovery from eucalyptus spent sulphite liquor by membrane processes. Biomass Bioenergy.

[175] Fernandes D.L.A., Silva C.M., Xavier A.M.R.B., Evtuguin D.V. (2012). Fractionation of sulphite spent liquor for biochemical processing using ion exchange resins. J. Biotechnol..

[176] Bhattacharya P.K., Todi R.K., Tiwar M., Bhattacharjee C., Bhattacharjee S., Datta S. (2005). Studies on ultrafiltration of spent sulfite liquor using various membranes for the recovery of lignosulphonates. Desalination.

[177] Madsen R.F., Nielsen W.K. (1978). Ultrafiltration of bleach effluents as an example of waste-water treatment by ultrafiltration. Desalination.

[178] Fernández-Rodríguez J., García A., Coz A., Labidi J. (2015). Spent sulphite liquor fractionation into lignosulphonates and fermentable sugars by ultrafiltration. Sep. Purif. Technol..

[179] Takahashi S., Tanifuji K., Shiell K., Fatehi P., Jahan M.S., Ohi H., Yonghao N. (2013). Removal of acetic acid from spent sulfite liquor using anion exchange resin for effective xylose fermentation with *Pichia stipitis*. BioResources.

[180] Alexandri M., Papapostolou H., Vlysidis A., Gardeli C., Komaitis M., Papnikolaou S., Kouticas A.A. (2016). Extration of phenolic compounds and succinic acid production from spent sulphite liquor. J. Chem. Technol. Biotechnol..

